# Integrator complex regulates NELF-mediated RNA polymerase II pause/release and processivity at coding genes

**DOI:** 10.1038/ncomms6531

**Published:** 2014-11-20

**Authors:** Bernd Stadelmayer, Gaël Micas, Adrien Gamot, Pascal Martin, Nathalie Malirat, Slavik Koval, Raoul Raffel, Bijan Sobhian, Dany Severac, Stéphanie Rialle, Hugues Parrinello, Olivier Cuvier, Monsef Benkirane

**Affiliations:** 1 Institute of Human Genetics, CNRS UPR1142, Laboratory of Molecular Virology; MGX-Montpellier GenomiX, 141 rue de la Cardonille, Montpellier 34396, France; 2 LBME-CNRS, Cell Cycle Chromatin Dynamics Laboratory. University Paul Sabatier, Toulouse 31061, France; 3 INRA, TOXALIM (Research Centre in Food Toxicology), Toulouse 31300, France; 4 IGF, MGX-Montpellier GenomiX, France

## Abstract

RNA polymerase II (RNAPII) pausing/termination shortly after initiation is a hallmark of gene regulation. Here, we show that negative elongation factor (NELF) interacts with Integrator complex subunits (INTScom), RNAPII and Spt5. The interaction between NELF and INTScom subunits is RNA and DNA independent. Using both human immunodeficiency virus type 1 promoter and genome-wide analyses, we demonstrate that Integrator subunits specifically control NELF-mediated RNAPII pause/release at coding genes. The strength of RNAPII pausing is determined by the nature of the NELF-associated INTScom subunits. Interestingly, in addition to controlling RNAPII pause-release INTS11 catalytic subunit of the INTScom is required for RNAPII processivity. Finally, INTScom target genes are enriched in human immunodeficiency virus type 1 transactivation response element/NELF binding element and in a 3' box sequence required for small nuclear RNA biogenesis. Revealing these unexpected functions of INTScom in regulating RNAPII pause-release and completion of mRNA synthesis of NELF-target genes will contribute to our understanding of the gene expression cycle.

## Abstract (web summary)

RNA polymerase II (RNAPII) pausing at transcriptional start sites is an important element of gene transcription regulation. Here, the authors implicate the Integrator complex as a regulator of RNAPII pause-release and completion of mRNA synthesis at a subset of the negative elongation factor target genes.

## Graphical abstract

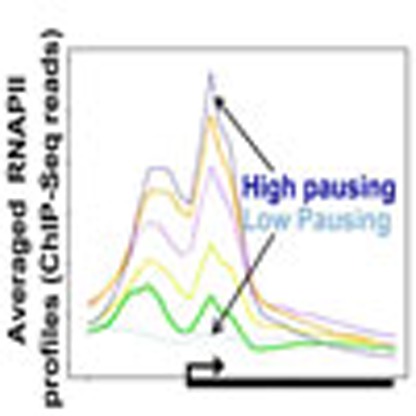

Promoter-proximal pausing of RNA polymerase II (RNAPII) is a negative regulatory mechanism of gene expression widespread in metazoans^1^. RNAPII pausing and elongation shortly after initiation is controlled through the action of negative and positive transcription elongation factors such as NTEF and P-TEFb, respectively^1^,^2^,^3^. NTEF consists of the negative elongation factor (NELF), a protein complex composed of four subunits (A, B, C/D and E) and the DRB (5,6-dichloro-1β-D-ribofuranosylbenzimidazole)-sensitivity-inducing factor DSIF, which is a heterodimer, composed of Spt4 and Spt5 (refs 4, 5). NELF and DSIF cooperatively establish RNAPII promoter-proximal pausing at least by binding to initiated RNAPII and possibly to the nascent transcript^5^,^6^,^7^. In order to overcome RNAPII pausing and to enter transcription elongation, P-TEFb is recruited to promoter-proximal region and phosphorylates NELF and Spt5 (refs 8, 9). Phosphorylated NELF dissociates from RNAPII while phosphorylation of Spt5 switches its activity from negative to positive elongation factor^10^. Additionally, P-TEFb phosphorylates the C-terminal domain (CTD) of the RNAPII largest subunit (RPB1) to ensure productive elongation^2^,^9^. RNAPII CTD, which consists of multiple heptad repeats of the consensus sequence YSPTSPS that can be phosphorylated at several sites, serves as a platform for the binding of factors required for the expression of RNAPII-transcribed genes^11^. Indeed, for protein coding genes P-TEFb-mediated phosphorylation of CTD at Serine 2 (Ser2) has been shown to have a key role in RNAPII pause-release and in coordinating/coupling transcription elongation to RNA processing, including splicing, 3'end processing and termination of transcription^11^,^12^. Phosphorylation of RNAPII CTD at Ser7 mediates the recruitment of Integrator complex (Integrator complex subunits (INTScom)), a ~14 subunit complex, to the promoter of small nuclear RNA (snRNA) genes to activate transcription and direct 3' end processing of the transcripts^13^,^14^.

A role for DSIF and NELF in inducing paused polymerase has been well established^3^. However, the reversal of RNAPII pausing does not always lead to the overexpression of target genes^15^. Indeed, NELF-depletion has only a limited effect on gene expression, possibly because NELF intrinsically has a limited effect on gene expression and/or because mRNA synthesis is a complex process involving multiple rate limiting steps which requires additional factors. Additionally, the strength of RNAPII pausing varies among genes. Thus, much remains to be learned about how the efficiency of RNAPII pausing and elongation is regulated at the mechanistic and biochemical level. In this study, we reveal a physical and functional link between NELF and INTScom. We show that INTScom is associated with TSS of NELF-target genes and that it regulates NELF-mediated RNAPII pausing. Interestingly, INTScom is also required for the production of mature mRNA from its target genes. Our study reveals an unexpected function of INTScom in regulating RNAPII pausing and processivity at coding genes.

## Results

### NELF interacts with the integrator complex subunits

**Figure 1:**
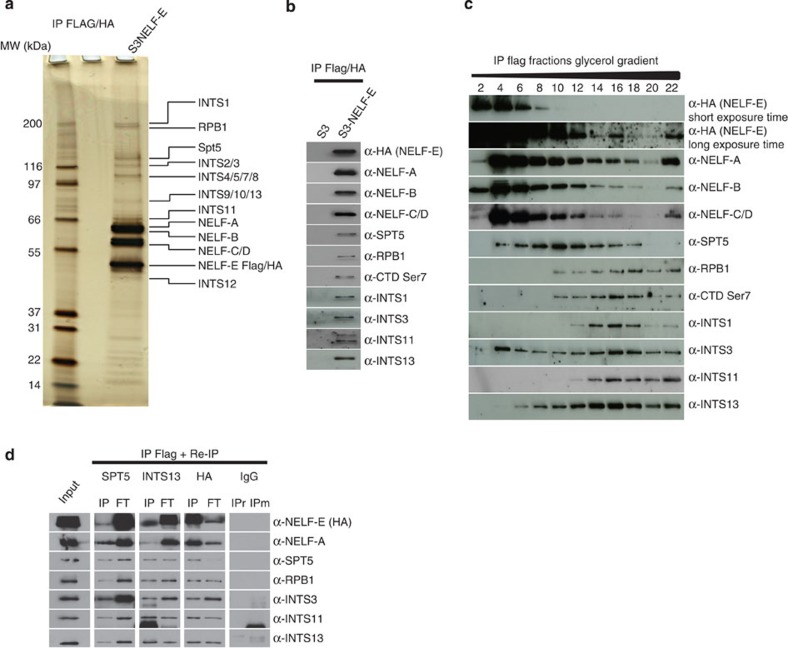
Immuno-purification of NELF. (**a**) Flag/HA-epitope-tagged NELF-E (eNELF-E) from HeLa S3 Dignam nuclear extracts was sequentially immunopurified on anti-Flag and anti-HA antibody-conjugated agarose beads. Purified material was separated by SDS–PAGE and visualised by silver staining. eNELF-E-associated proteins were identified by MS (see Supplementary Dataset 1). (**b**) Flag/HA IPs from samples shown in (**a**) were separated by SDS–PAGE and the presence of eNELF-E-associated proteins identified was confirmed by immunoblotting. (**c**) Glycerol gradient sedimentation analysis of eNELF-E. Flag-purified eNELF-E-associated complexes were separated by centrifugation through a 12–40% glycerol gradient. Material of even-numbered fractions was resolved by SDS–PAGE and probed for identified proteins. (**d**) Reciprocal IPs (ReIPs): Flag-purified eNELF-E (Input) was subjected to IP using anti-Spt5, anti-INTS13, anti-HA antibodies or irrelevant rabbit IgG (IPr) or mouse IgG (IPm). Input, IP, as well as flow through (FT) were probed for eNELF-associated proteins.

To gain more insight into NELF-mediated RNAPII pausing we immunopurified (IP) NELF and its associated partners from HeLa nuclear extracts. Dignam nuclear extracts prepared from control and Flag-HA-tagged NELF-E subunit (eNELF-E) expressing cells were subjected to tandem affinity chromatography as previously described^16^. Flag-HA IPed materials were run on SDS–polyacrylamide gel electrophoresis (SDS–PAGE) and proteins were visualised by silver staining and identified by mass spectrometry (MS) (Fig. 1a and Supplementary Dataset 1). Major MS-identified eNELF-E nuclear partners are the core subunits of the NELF complex (A, B, C/D). As previously reported, both Spt5 and subunits of RNAPII were recovered^4^. Interestingly, MS identified all subunits of the Integrator complex (INTScom) (Fig. 1a). We first confirmed the interaction between INTScom subunits and NELF-E (Fig. 1b). As negative controls, subunits of the NURD and INO80 complexes were absent in eNELF-E immunopurified materials (Supplementary Fig. 1A). Interestingly, we found that RPB1 associated with eNELF-E is phosphorylated on Ser7 but not on Serine 2 or Ser5 (Fig. 1b and Supplementary Fig. 1A). We next asked whether the interaction between NELF and INTScom subunits is mediated through nucleic acids or by protein–protein interactions. For this purpose, nuclear extracts were treated with either RNase or DNase and Ethidium bromide prior IP using anti-Flag antibody. The presence of INTS3 and INTS11 subunits in IPed material was assessed by western blotting. We found that NELF/INTScom interaction is independent of RNA and DNA (Supplementary Fig. 1B). As positive control, RNase treatment resulted in reduced interaction between CyclinT1, CDK9 and HEXIM (Supplementary Fig. 1C). Thus, and in agreement with the recent finding by Yamaguchi and colleagues^17^, NELF/INTScom interaction is mediated through protein–protein interaction. To test whether NELF, INTScom, Spt5 and RPB1 associate in a single complex, glycerol gradient sedimentation of Flag-purified eNELF-E was performed (Fig. 1c). The presence of eNELF-E-interacting proteins in the collected fractions was analysed by western blot. Fig. 1c shows a major peak containing NELF and the INTS3 subunit (fraction 4) and a second peak containing NELF, INTScom and RPB1 (Fraction 16). To further characterise NELF-associated proteins, Flag IPed NELF-E was subjected to second IPs (ReIP) using anti-Spt5, anti-INTS13 and anti-HA antibodies. As shown in Fig. 1d, a small fraction of subunits of the INTScom, NELF-A, NELF-E, Spt5 and RPB1 were present in ReIP using anti-Spt5 and anti-INTS13 antibodies suggesting that a fraction of NELF associates with INTScom, possibly as part of a RNAPII complex on the gene. Altogether, these experiments suggest that NELF interacts with INTScom and with INTS3 independently of other INSTcom subunits.

### INTScom regulates RNAPII pausing and processivity at the HIV-1 LTR

**Figure 2:**
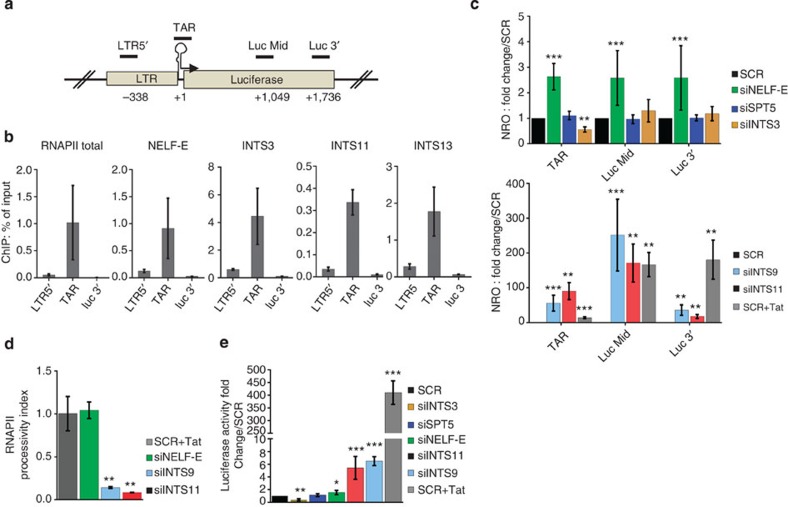
Integrator complex regulates transcription elongation at the HIV-1 LTR. (**a**) Schematic representation of the LTR-Luciferase locus in HeLa-LTR-*Luciferase* cells depicting positions of primers used in ChIP and NRO (Nuclear Run-On) assays. (**b**) ChIP was performed using indicated antibodies and chromatin prepared from HeLa-LTR-*Luc* cells. Enrichments are presented as percentage of input. (**c**) NROs were performed using nuclei prepared from HeLa-LTR-*Luc* cells transfected with the indicated siRNAs. Enrichment of mRNA containing Br-UTP after IPs is shown as Supplementary Fig. 3B. Values were normalised to the amount of 4 control RNAs (RPS14, 7SK, KDSR and PIGB; see Supplementary Fig. 3C) in the same samples. Results are presented as fold change over control condition SCR, the average profiles of the four normalisations are shown. Knockdown efficiencies of siRNAs were assessed by immunoblotting (Supplementary Fig. 3A). (**d**) RNAPII processivity index calculated as the ratio of fold change luc3' to fold change lucMid from NROs shown in (**c**). Results are statistically compared to the positive control condition SCR+Tat. (**e**) Luciferase activity in HeLa-LTR-*Luciferase* cell extracts monitoring HIV-1 LTR activity on protein level in indicated knockdown backgrounds. Results are presented as fold change over control condition SCR. *P-value<0.05; **P-value<0.005; ***P-value<0.0005, no*=no significant *P*-value as measured by student’s *t*-test. Error bars represent standard deviations (*n*=3).

INTScom is a large multisubunit complex known to associate with the CTD of RPB1 and to catalyse the endonucleolytic cleavage of nascent snRNAs near their 3' ends^13^,^18^,^19^. To explore a role for the INTScom in NELF-mediated RNAPII pausing at protein coding genes, we used the human immunodeficiency virus type 1 (HIV-1) promoter. Transcription from the HIV-1 long terminal repeat (LTR) leads to RNAPII pausing and premature termination after synthesis of a short RNA, the transactivation response element (TAR)^8^,^20^,^21^. We first asked whether INTScom subunits, like NELF associate with the viral LTR. Chromatin immunoprecipitation (ChIP) was performed using chromatin prepared from HeLa cells containing a single copy of an integrated LTR-*luciferase* reporter gene (HeLaLTR-*luc*) and the indicated antibodies. INTScom subunits accumulate where expected on U2 snRNA chromatin (Supplementary Fig. 2). As expected, NELF and RNAPII were found at the promoter-proximal region (TAR) (Fig. 2a,b) of the integrated LTR-*luc* reporter construct. Like NELF and RNAPII, INTS3, INTS11 and INTS13 associate with the TAR region and were absent from the 5'upstream region and the 3' end of the luciferase reporter (Fig. 2a,b). This experiment shows that INTScom subunits associate with the promoter-proximal region of the HIV-1 LTR. We next analysed the role of Integrator subunits in regulating transcription from the viral LTR by nuclear run-on assay (NRO). HeLaLTR-*luc* were transfected with siRNA specific for INTS3, INTS9 or INTS11. Non-targeting scrambled (SCR) siRNA, NELF-E or Spt5-specific siRNAs were used as controls (see Supplementary Fig. 3A for knockdown efficiency). Treatment of SCR-transfected cells with the viral transactivator Tat was used as positive control. As shown in Fig. 2c (upper panel), knockdown of NELF-E, but not Spt5, results in transcriptional activation of the reporter construct. Knockdown of INTS3, like that of Spt5, resulted in no obvious transcription phenotype (Fig. 2c, upper panel). Interestingly, knockdown of the catalytic INTS11 subunit and its regulatory subunit INTS9 results in a dramatic transcriptional activation of the viral LTR (Fig. 2c, lower panel). However, and in contrast to transcriptional activation by the viral transactivator Tat (Fig. 2c, lower panel), such increase in transcription was largely observed up to the middle of the reporter construct and highly limited at the 3' end of the reporter (Fig. 2c, lower panel). Consistently, the RNAPII processivity index was five times lower in INTS11 and INTS9 knockdown cells as compared to cells knocked down for NELF-E or treated with Tat (Fig. 2d). In addition, a correlation between transcriptional activation (Fig. 2c) of the reporter construct and luciferase assay is observed in Tat and siNELF-E treated cells (Fig. 2e). Only a 4–6-fold induction of luciferase activity is observed in siINTS11 and siINTS9-treated cells (Fig. 2e) contrasting with their strong impact on transcriptional activation. Consistently, when the LTR-*luc* reporter mRNA (polyadenylated) was quantified by reverse transcriptase-quantitative PCR, we observed a modest increase of the reporter mRNA in INTS11-KD cells (Supplementary Fig. 3D). Of note, in this assay knockdown of INTS11 had no effect on TAR region (Supplementary Fig. 3D) suggesting a role of INTS11 in regulating transcription elongation at the viral promoter. Taken together, our results revealed an unexpected dual function of the INTScom in regulating RNAPII pausing and processivity at the HIV-1 promoter.

### INTScom dictates the strength of NELF-mediated RNAPII pausing

**Figure 3:**
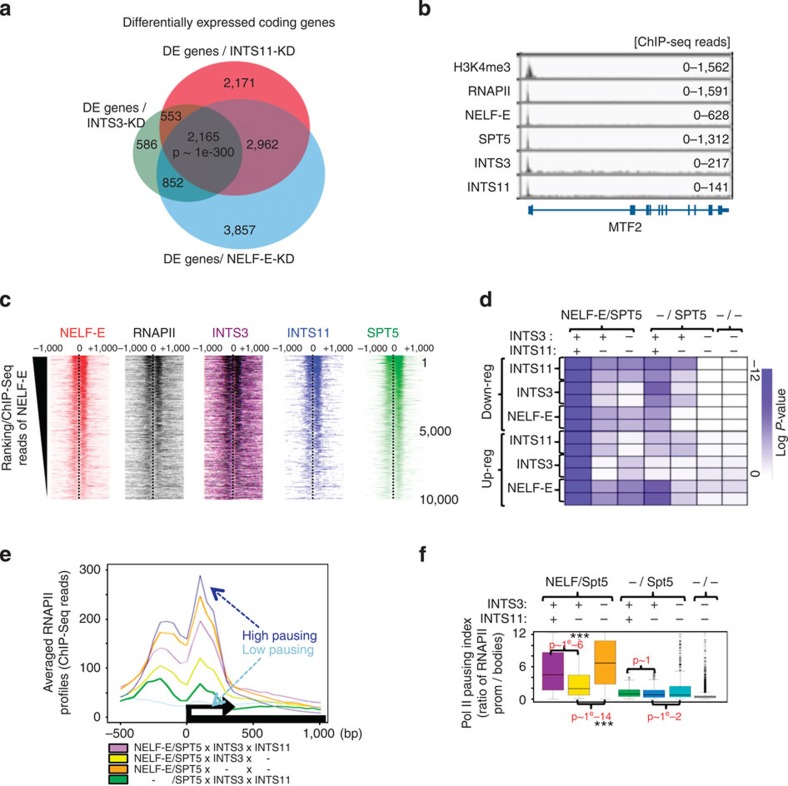
Integrator complex regulates NELF-mediated RNAPII pausing at coding genes. (**a**) Venn diagram showing the intersection among differentially expressed (DE) genes identified by microarray analysis (*n*=3) upon depletion of −INTS11, −INTS3− or −NELF-E (see Methods; *P*<0.001). (**b**) Genomic view of ChIP-Seq data over the protein coding *MTF2* gene for: H3K4me3, total RNAPII, NELF-E, SPT5, INTS3 and INTS11. (**c**) Heat maps showing the binding of NELF-E, RNAPII, INTS3 and INTS11 by ranking the top 10,000 genes according to NELF binding (+/− 250 bp from TSSs; see Methods). (**d**) Heat map showing the intersection analyses between DE (down- or up- regulated) genes and the corresponding TSS bound or not (+/−) by NELF-E, INTS3 and INTS11 (see Methods). Differential expression (DE) was analysed in R using limma (adjusted *P*<0.01; see Supplementary Dataset 2B–D; see Methods). The enrichment between these two lists is indicated in Log *P*-value (see colour bar). (**e**) Averaged RNA RNAPII profiles as measured by ChIP-Seq as a function of NELF-E-, Spt5-, INTS3 and INTS11 binding. The ChIP-Seq profiles of ‘High/Low pausing’ are indicated for comparison (RNAPII profiles of highly paused (blue line)- or active genes (light blue line), respectively, as previously described^22^). Genes bound by −NELF, SPT5 and INTS3 with or without INTS11 (purple and orange line, respectively) or by −NELF and SPT5 with or without INTS3 (yellow and orange line, respectively) or by SPT5, INTS3 and INTS11 (green line). (**f**) Box plots showing the RNAPII pausing indices depending on NELF/SPT5/INTS3 and INTS11 binding or not (+/−) to the corresponding TSS. *P*-values, Wilcoxon pair-wise test (see also Supplementary Fig. 7A).

We next asked whether the function of the INTScom in regulating RNAPII pausing at the HIV-1 promoter also applies to cellular coding genes. Supporting this idea, microarray analyses showed that INTS3 and INTS11depletion (see Supplementary Fig. 4A for knockdown efficiency) from HeLa cells led to changes in the expression of thousands of genes (Fig. 3a and Supplementary Dataset 2a–d). The impact on gene expression could not be attributed to the loss of U1 or U2 snRNA, since no significant changes in their expression were observed within the time frame of these experiments (data not shown) or to an effect on cell cycle (Supplementary Fig. 4B). Interestingly, a majority of these genes (2,164 genes) were commonly identified as differentially expressed (DE) upon depletion of NELF (Fig. 3a and Supplementary Dataset 2a). Such significant overlap (*P*-value <1e−300) highlighted a novel possible function of INTScom subunits in co-regulating NELF-dependent coding genes.

To further investigate whether this involves a direct role of INTScom subunits, ChIP coupled to high-throughput sequencing (ChIP-Seq) was performed using antibodies specific for NELF-E, Spt5, INTS3, INTS11 and H3K4me3. The identified ChIP-Seq peaks of INTS11, like NELF-E, preferentially localised close to the TSSs of coding genes (+/− 250 bp) and INTS3 showed similar distributions to Spt5 (Fig. 3b,c Supplementary Fig. 5A and Supplementary Dataset 3). Heat maps of the ChIP-Seq reads organised by ranking of genes according to NELF-E levels showed a tight correlation between its binding and the binding of INTScom subunits near TSSs (Fig. 3c), as confirmed by analysis of the averaged distribution of their ChIP-Seq peaks (Supplementary Fig. 5C,D). Moreover, intersection analyses of ChIP-Seq data showed that the binding sites of INTS3 and INTS11 tightly overlapped with that of NELF and Spt5 (Supplementary Fig. 5B; *P*-value <1e−300). INTS11 binding sites were found provided INTS3 was also bound with very few exceptions (16 genes). Accordingly, clustering analyses recapitulated the tight correlations found amongst NELF/Spt5, RNAPII pausing and INTScom subunits with respect to their individual binding intensities to each TSS, at genome-wide levels (Supplementary Fig. 6). As such our results highlight that the recruitment of INTScom to TSS is correlated to the presence of NELF-Spt5. Finally, direct targets bound by INTS3/11 and NELF-E/Spt5 were highly enriched among the differentially regulated genes upon INTS-3/11 depletion in microarray analysis (Fig. 3d; *P*<1e−12; Supplementary Dataset 2a–d), showing a direct function of INTScom subunits in co-regulating the expression of protein coding genes together with NELF/Spt5.

Taken together, our data raised the hypothesis that INTScom subunits impact the expression of their direct target genes through the regulation of NELF-mediated RNAPII pausing. In support of this hypothesis, inspection of averaged RNAPII ChIP-Seq profiles over TSS (Fig. 3e) and calculation of the ‘pausing index’ (Fig. 3f) showed that the presence of INTS11 and/or INTS3 correlates to RNAPII accumulation around the TSS (Fig. 3e) and NELF/Spt5-mediated RNAPII pausing (Fig. 3f). Indeed, genes containing NELF, Spt5 and INTS3 show lower RNAPII occupancy around the TSS and a lower pausing index as compared with genes bound by NELF, Spt5, INTS3 and INTS11 or to genes containing NELF and Spt5 only (Fig. 3e–f; *P*-value ~1e−6 and −14, respectively). The correlation of INTS3 and INTS11 on RNAPII pausing was most significant in the context of paused genes that are bound by NELF (Fig. 3f, compare box 1 with boxes 2 and 3, or 4 with 5 and 6, respectively). Such analysis also suggested that INTS3 and INTS11 may have opposite effects on NELF-mediated RNAPII pausing or elongation (Supplementary Fig. 7A). Of note, calculation of the pausing index confirmed the highly specific correlation between NELF binding and RNAPII pausing (Supplementary Fig. 7B), validating our analyses^22^.

**Figure 4:**
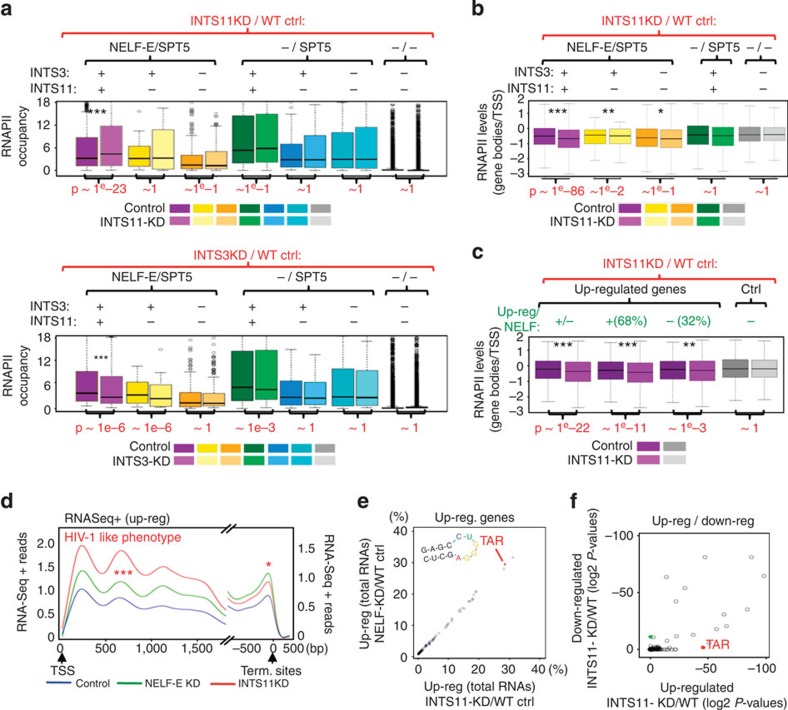
Integrator regulates RNAPII occupancy and processivity of NELF-target genes. (**a**) Box plots showing the variations in RNAPII occupancy (ChIP-Seq reads over gene bodies; see Methods) between control and INTS11- (upper panel) or INTS3- (lower panel) -depleted cells (see Supplementary Fig. 4A for the knockdown efficiency) as a function of NELF-E/Spt5, INTS3 and/or INTS11 binding or not (as indicated on top). *P*-values, wilcoxon pair-wise tests. (**b**) Box plot showing RNAPII occupancy over gene bodies normalised to TSSs (see Methods) between control and INTS11-depleted cells depending on NELF-E/Spt5, INTS3 and/or INTS11 binding or not (as indicated on top). *P*-values, pair-wise wilcoxon tests. (**c**) Box plots showing the variations in RNAPII occupancy (see panel B) upon INTS11-KD for groups of up-regulated genes (upon INTS11-KD and/or of NELF-KD) and control genes (no change in expression; boxes in grey). *P*-values, wilcoxon pair-wise tests. 68/32%, percentages of genes up-regulated upon INTS11-KD that were also up-regulated upon NELF-KD (68%) or not (32%)(see Fig. 3a; Supplementary Dataset 2 for a list). (**d**) Average profiles of RNASeq ‘+’ reads in control-, INTS11− or NELF-E- depleted cells over exons (left) or transcription ends (right) for ‘direct targets’ (genes bound by NELF-E/INTS3/INTS11) and that were up-regulated upon INTS11-KD as compared to control. *Y* axis, normalised ChIP-Seq reads (see Methods). (**e**) Scatter plot showing the percentage of INTS11 (*x* axis) and of NELF (*y* axis) ChIP-Seq peaks harbouring a given consensus motif. Binding motifs were obtained through systematic search among all ChIP-Seq peaks of INTS11 and NELF using RSAT (see Methods). Only the motifs showing significant intersections with INTS11 or NELF peaks (fisher exact test<1e−3) were analysed (see Supplementary Dataset 5 for a list). (**f**) Scatter plot showing the enrichment of genes harbouring a given motif (+/− 250 bp from TSSs; see Methods) among DE genes (up- or down- regulated upon INTS11-KD; *x* axis and *y* axis, respectively; in Log2 *P*-values). Note that TAR motif are enriched among up-regulated genes (see Supplementary Dataset 5 for a list of all motifs).

To further assess how INTS3/11 may influence RNAPII occupancy, ChIP-Seq analyses of RNAPII were performed upon depletion of either subunit (see Supplementary Fig. 7C for knockdown efficiency). INTS11 depletion increased RNAPII occupancy over gene bodies (Fig. 4a), that is, the number of ChIP-Seq reads found in gene bodies (see Methods). Such increase in reads was specific for direct targets whose promoters were bound by NELF and INTS3 (Fig. 4a, upper panel; compare boxes 1 and 2 with 6 and 7; *P*-values of 1e−23 and 1, respectively). In stark contrast, INTS3-depletion led to the opposite effect, namely the decrease of RNAPII occupancy over NELF-regulated genes (Fig. 4a, lower panel, *P*~1e−6 and 1, respectively). Further normalisation of ChIP-Seq reads in gene bodies by reads over TSSs showed a specific decrease of RNAPII occupancy for the subset of genes that are bound by INTScom subunits and NELF (Fig. 4b). Such decrease was detected for the genes that were up-regulated upon INTS11-KD and NELF-KD (Fig. 4c).

We next asked whether the impact of INTS11 on RNAPII occupancy might in turn affect RNA levels over its target genes. Inspection of the averaged profile of strand-specific RNASeq (+) reads showed a significant increase along the bodies of up-regulated genes upon INTS11-KD (Fig. 4d; *P*~1e−12; Supplementary Fig. 7d; left box plot and Supplementary Dataset 4). Of interest, the variation in read counts between INTS11-KD and control was barely significant over termination sites (Fig. 4d; Supplementary Fig. 7d; *P*~1e−2; right box plot). Such ‘HIV-like’ phenotype was highly enriched among ‘direct targets’ (genes bound by NELF, Spt5, INTS3 and INTS11) corresponding to a total of 859 genes. Approximately half of these genes (432 genes; *P*~1e−191) were further down-regulated upon INTS3-KD in complete agreement with the antagonistic effect of INTS3 and INTS11 (Fig. 4a; Supplementary Fig. 7a). Thus, similarly to the HIV promoter (Fig. 2), these results support a role of INTScom in regulating RNA processing at coding genes.

### INTScom target genes are enriched in hNBE and 3'box sequences

We next analysed whether the INTScom regulated coding genes share some sequence determinants. Systematic motif search from the ChIP-Seq peaks of NELF or INTScom subunits INTS11/3 showed that the binding of INTS11 tightly correlated with that of NELF independently of the subsets of promoters analysed (Fig. 4e; Supplementary Fig. 8a). No motif could be identified that might account for the recruitment of INTScom independently of NELF. This is in complete agreement with our genome-wide analyses showing that INTScom is preferentially recruited to NELF-target genes (Supplementary Fig. 5B). Of interest, TSSs of cellular genes up-regulated upon INTS11-KD were specifically enriched in the HIV-1 TAR/loop motif recently identified as the human NELF Binding Element (‘hNBE’^23^) (Fig. 4e ‘CUGGGA’ and Supplementary Dataset 5). Accordingly, TSSs harbouring such a motif were highly enriched in the group of genes that were up-regulated upon INTS11-KD (Fig. 4f, *P*-value~1e−48; Supplementary Fig. 8b), by contrast to down-regulated genes. Our data shows that hNBE/TAR motif is a marker for INTS11 target genes, reinforcing the functional link between INTS11 and NELF in regulating RNAPII pausing at coding genes.

**Figure 5:**
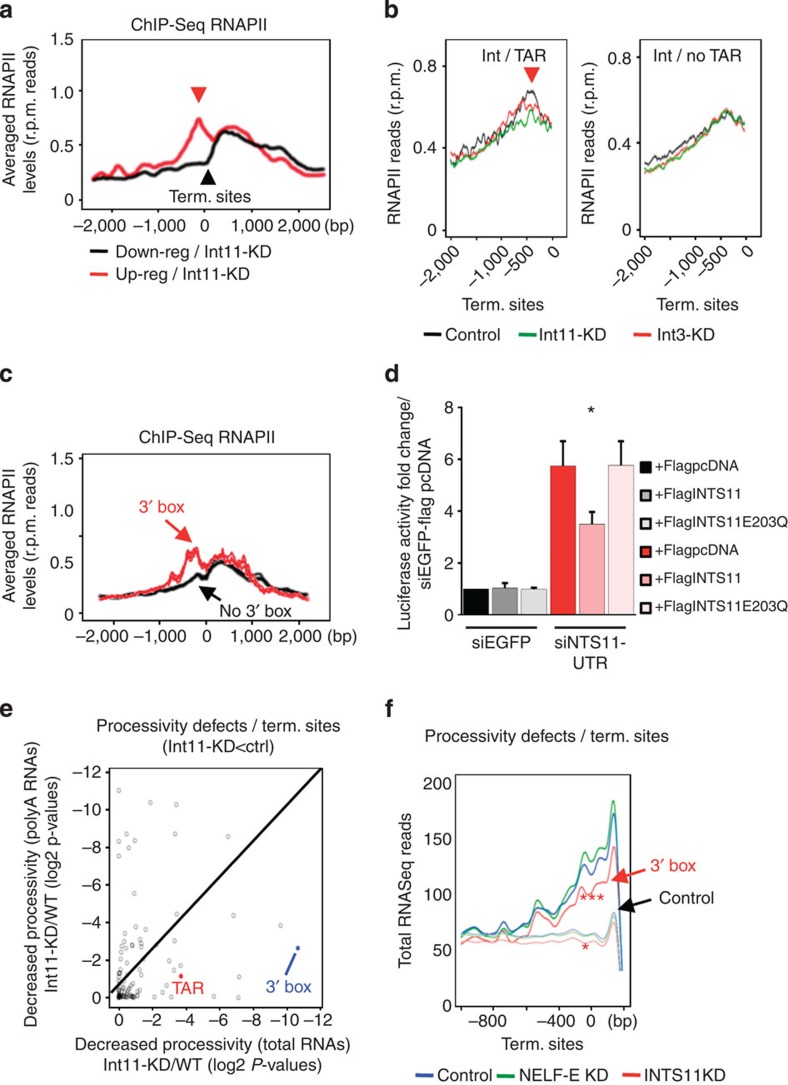
Integrator regulates RNAPII occupancy at transcription termination sites (TES) of its target genes. (**a**) Averaged RNAPII profiles (*y* axis) ChIP-Seq reads) +/− 2000, bp of TES (*x* axis) for up- (red plot) or down- (black plot) regulated genes (INTS11-KD as compared to control cells). Red arrow indicates RNAPII peak. (**b**) Averaged RNAPII profiles (*y* axis) near TES (*x* axis) in knockdown or control cells and for INTScom target genes harbouring (left) or not (right) TAR motif (+/− 250 bp from TSS). Variation in read counts was scored for TAR-containing (*P*~1e−85; pair-wise wilcoxon test) compared to no TAR-containing genes (*P* ~1). (**c**) Averaged RNAPII profiles of TES (*x* axis) corresponding to genes harbouring (red plot; >1,200 genes; see Supplementary Dataset 5) or not (black plot) a 3' box near their TES. (**d**) Luciferase activity, in extracts of HeLa-LTR-Luciferase cells transfected with control siRNA (siEGFP) or siRNA targeting INTS11UTR and indicated plasmids, shown as fold change over control siEGFP+FlagpcDNA. KD and overexpression efficiencies are shown as Supplementary Fig. 8C. *P-value<0.05; **P-value<0.005; ***P-value<0.0005, no*=not significant as measured by student’s *t*-test. Error bars represent standard deviations (*n*=3). (**e**) Scatter plot scoring the variations in RNASeq reads near TES (normalised to gene bodies) between INTS11-KD and control. The variations in processivity were quantified as log *P*-values (x and y axes) scoring variations for every group of genes depending on the presence or not of a DNA motif close to their TSSs or to their TES (see Methods). Variations were measured for total RNAs (*x* axis) and for polyA+ RNAs. The red and blue dots correspond to the scores obtained for the subset of genes harbouring TAR or 3' box (see Supplementary Dataset 5). (**f**) Averaged RNASeq+ reads in knockdown or control cells (as indicated) near TES (*x* axis) of genes bound by INTScom harbouring a 3' box (red plot; >1,200 genes; see Supplementary Dataset 5) or not (‘control’ genes) near their TES.

Inspection of RNAPII profiles over the termination sites of the 859 ‘direct targets’ of INTS11 (up-regulated genes upon INTS11-KD and with INTScom/NELF peaks in their promoters) showed that they harboured a RNAPII peak within the last 500 bp before termination sites (Fig. 5a; see red plot). By contrast, such termination site-associated (‘TESA’) RNAPII peak was not detected near the termination sites of down-regulated or control genes upon INTS11-KD (Fig. 5a; see black plot). Interestingly, the TESA RNAPII peak was also detected for the thousands of promoters bound by INTScom provided that the hNBE/TAR motif was also present (Fig. 5b; compare left and right plots, black curves). In complete agreement, the promoters of up-regulated genes are specifically enriched in hNBE/TAR motifs (Fig. 4f). Also, INTS11-KD decreased significantly the averaged RNAPII levels in the region corresponding to the TESA peak, as compared to control cells (Fig. 5b, compare green and black curves; *P*-values +/− TAR:~1e−48 and 1e−3, respectively). Therefore, our data support the view that similar to HIV promoter, presence of an hNBE/TAR close to paused sites may be prone to INTScom-mediated control of RNA processing.

INTScom-mediated U2/snRNA processing depends on the presence of a 3' box (‘AAAAACAGACC’) upstream of the termination site^14^,^24^. Importantly, we found a 3' box close to termination sites (<1,500 bp) in more than 1,200 coding genes (see Supplementary Dataset 5). Also, nearly 40% of the genes bound by INTScom and NELF also contained such 3' box near their termination sites (834/2,127). Of the INTScom target genes, only those also harbouring this 3' box showed a clear TESA RNAPII peak, in stark contrast to those with no 3' box near their termination site (Fig. 5c; compare red and black plots, respectively). As such, these results raised the possibility that, similar to snRNAs, 3'box sequence exacerbates INTScom-mediated RNAPII processivity defects at a subset of its target coding genes. We therefore asked whether INTS11 endonuclease activity plays a role in regulating RNAPII processivity. Complementation experiments were performed using HeLaLTR-luc cells knocked down for INTS11. We found that wild type INTS11, but not INTS11E203Q catalytic mutant, significantly reverted the enhanced luciferase expression after endogenous INTS11-KD (Fig. 5d and Supplementary Fig. 8C), suggesting that the endonuclease activity of INTS11 is required for RNAPII pausing and processivity at the HIV-1 promoter.

### INTScom is required for production of mature mRNA

To extend our results showing that INTScom is required for production of full length transcript from the HIV reporter construct to its target genes, we measured the relative variations of RNASeq reads over termination sites (−500 to 0) normalised to gene bodies, for INTS11-depleted cells as compared to control cells. These measures were performed both for total RNAs as well as for mature RNAs pre-selected through polyA+ purification (see Methods). Such analysis showed that INTS11-KD led to a decrease of RNA levels near 3' ends specifically for genes bound by INTScom and NELF (Supplementary Dataset 6A,B) as well as for genes harbouring hNBE/TAR motif near their TSSs (Supplementary Fig. 8D,E). Of interest, the decrease in RNA levels at termination sites was not detected using mature RNAs (Supplementary Fig. 8F,G; compare left and right plots/box plots), supporting a role of INTS11 prior to production of mature polyadenylated RNAs, in complete agreement with our data using HIV reporter (Fig. 2).

Systematic scoring of RNASeq reads near termination sites between INTS11-KD and control, for total RNAs or polyA+ RNAs (see Methods) allowed us to further highlight the role of INTS11 in RNA processivity. The scored defects correspond to genes harbouring decreasing levels of reads near termination sites with respect to reads over gene bodies, similarly to what was found by the NRO. Genes harbouring such defects upon INTS11-KD largely overlap with those showing RNA processivity defects upon NELF-KD (Supplementary Fig. 8H; *P*-value~1e−206), reinforcing the functional interaction between NELF and INTScom. Processivity defects are also observed by normalising RNAPII ChIP-Seq reads near termination sites with reads in gene bodies (Supplementary Fig. 8I), as detected for the list of down-regulated genes upon INTS11-KD. Of interest, the 3' box was one of the most specific motifs associated with RNA processing defect at the termination sites upon INTS11-KD (Fig. 5e). Also, the presence of a 3' box is specifically associated with decreased levels of total RNAs in INTS11-KD as compared with control cells with no significant variations in mature RNA levels (Fig. 5e; *P*-value~1e−11 and 1e−1, respectively). Accordingly, comparison of RNASeq profiles between INTS11-KD and control cells showed that the presence of a 3' box was required to detect a specific decrease in RNA levels over termination sites (Fig. 5f; *P*-value~1e−105), in stark contrast to what was found for mature RNA levels (Supplementary Fig. 8J; *P*-value~1). Our data highlighting the INTS11-mediated regulation of RNA levels near termination sites, in the presence of a 3' box, suggest a new role of INTScom in regulating RNA processing of coding genes. Of interest, genes harbouring such processing defects are largely enriched within gene ontologies involved in responses to various stresses (Supplementary Fig. 9), supporting the link between INTScom and NELF-mediated RNAPII pausing.

INTS11-KD also led to an increase of antisense RNASeq ‘−’ reads (Supplementary Fig. 10A) as evidenced by strand-specific RNASeq analysis. Heat map showing the net variations in RNASeq ‘−’ reads between INTS11-KD and control cells (see Methods) highlighted that the increased levels in antisense reads tightly correlated with INTS11-mediated regulation of coding genes (Supplementary Fig. 10B), as illustrated by the WDR75 gene (Supplementary Fig. 10C). Thus, INTScom regulates RNAPII-mediated divergent transcription of coding genes.

## Discussion

Our study reveals an unexpected function of INTScom in regulating both RNAPII pause-release and processivity of NELF-target genes. Biochemical analyses show that a fraction of NELF associates with INTScom, RNAPII and Spt5. The observed interaction between NELF and INTScom is mediated through protein–protein interaction. This is consistent with the recent report showing that, *in vitro*, recombinant NELF produced in bacteria, particularly GST-NELF-A, binds to the recombinant INTScom produced in insect cells^17^. In the same study, GST-CTD is shown to interact with recombinant INTScom only when CTD is phosphorylated by P-TEFb. Interestingly, we found that RPB1 associated with NELF/INTScom is phosphorylated on Ser7, suggesting its role in the assembly of the complex. Taken together, both studies (ref. 17 and our manuscript) converge towards a model where INTScom establishes interactions with both NELF and RPB1. An interesting observation from our biochemical study is the fact that INTS3 is able to interact with NELF and Spt5 in the absence of the other INTScom subunits and RNAPII. This is consistent with previous reports showing that INTS3 can achieve cellular functions independently of other INTScom subunits through interactions with other cellular complexes^25^,^26^,^27^,^28^. Both biochemical and functional analyses show that the strength of RNAPII pausing mediated by NELF depends on whether the genes are bound by NELF-Spt5, NELF-Spt5-INTScom or NELF-Spt5-INTS3 ranging from highly paused to low-paused genes, respectively.

An intriguing observation is the opposite activity of INTS3 and INTS11 on NELF-mediated RNAPII pausing. The presence of INTS3 alone correlates with low RNAPII density at the TSS and increased RNAPII in gene bodies. In complete agreement, its depletion reduces RNAPII occupancy over gene bodies. By contrast, INTS11 depletion increases RNAPII occupancy and RNA levels over gene bodies but not over termination sites, resulting in defects in RNAPII processivity and RNA processing. This is in agreement with the recent report showing that INTScom is required for the recruitment of the super elongation complex to ensure a productive transcription elongation^29^. As such, INTScom subunits may couple the release from NELF-mediated RNAPII pausing to RNA processing. How INTScom is recruited to NELF-target genes is unknown. That NELF, Spt5, INTScom and RNAPII interaction is independent of nucleic acids suggests a direct recruitment as a complex to target genes. However, it is possible that they assemble as a complex at the TSS of NELF-target genes. Nevertheless, INTScom-mediated regulation of coding genes is restricted to NELF-target genes. This is consistent with the fact that INTScom target genes are highly enriched in hNBE motif around the TSS^23^ (Fig.4e). Our data suggest that the catalytic activity of INTS11 may play a role in regulating RNAPII processivity. Interestingly, our sequence motifs search revealed the presence of the 3' box sequence close to termination sites of INTScom target coding genes. The presence of a 3' box was associated with a decrease in RNA levels over termination sites in INTS11-depleted cells. These data suggest a functional contribution of the 3' box in INTS11-mediated regulation of RNA processing. INTScom may thus play a key role in allowing NELF to couple the pause-release of RNAPII to mRNA processing^12^. Deciphering the mechanism by which INTScom regulates NELF-target genes will certainly make an important contribution to our understanding of the transcription cycle.

## Methods

### Immuno-purification of NELF-E

NELF-E cDNA was purchased from Source BioScience LifeSciences (Clone IRAUp969C0381D) and cloned into pOZ-FH-N^16^. NELF-E was purified from Dignam nuclear extracts^30^ derived from HeLa S3 cells stably expressing Flag- and HA-tagged NELF-E (eNELF-E) by two-step affinity chromatography^16^. Nuclear extracts were first incubated with anti-FLAG antibody-conjugated agarose beads (see list of antibodies in Supplementary Methods) and the bound polypeptides were eluted with FLAG peptide (Sigma) under native conditions. The FLAG affinity-purified material was further immunopurified by affinity chromatography using anti-HA antibody-conjugated agarose beads (see list of antibodies) and eluted under native conditions using HA peptide (Roche). Five percent of FLAG and HA immunoaffinity-purified eNELF-E or mock immunoprecipitations from 4 l of culture were resolved on SDS–PAGE and stained with the Silverquest-kit (Invitrogen). The remainder of the eluate was stained with Coomassie-R250. Individual Coomassie-R250 stained bands or for closely migrating bands, regions of the gel, were excised and subsequently analysed by tandem MS at the Harvard Medical School Taplin Biological Mass Spectrometry facility, Boston, MA, USA.

### Glycerol gradient sedimentation analysis

For density gradient sedimentation, 100 μl of FLAG-purified material was loaded onto a 4.5 ml 12–40% glycerol gradient in buffer G (20 mM Tris-HCl [pH 7.5], 150 mM NaCl, 5 mM MgCl2, 0.1% Tween 20, 10 mM beta-mercaptoethanol, 0.5 mM PMSF) and centrifuged for 4 h at 55000, r.p.m. in a Beckman SW55Ti rotor (Beckman coulter). 200 μl fractions were collected from the top of the gradient.

### Cloning of Integrator complex subunit 11

Integrator 11 (INTS11) cDNA was purchased from Source BioScience LifeSciences (Clone IRAUp969B0740D). INTS11E203Q was a kind gift of Yamaguchi Y. Open reading frames were cloned into the pcDNA vector and a Flag-tag was added to the N-termini.

### Chromatin immunoprecipitations

ChIPs were performed as described in Whyte *et al.*^31^ using ~100 × 10^6^ HeLa-LTR-Luciferase cells as starting material with the exception of an additional nuclei purification step. Briefly, cells were thawed and resuspended in 1 ml Buffer A (0.3 M SUCROSE, 60 mM KCl, 15 mM NaCl, 5 mM MgCl, 0.1 mM EGTA, Tris-HCl pH=7.5, 0.2 mM PMSF), 1 ml Buffer B (0.3 M SUCROSE, 0.2% NP40, 60 mM KCl, 15 mM NaCl, 5 mM MgCl, 0.1 mM EGTA, Tris-HCl pH=7.5, 0.2 mM PMSF) was then added and incubated for 7 min on ice, laid over a 8 ml cushion of Buffer C (1.2 M SUCROSE, 0.2% NP40, 60 mM KCl, 15 mM NaCl, 5 mM MgCl, 0.1 mM EGTA, Tris-HCl pH=7.5, 0.2 mM PMSF) and spinned for 20 min at 3000, r.p.m. on 4 °C. Pelleted nuclei were resuspended in 3-ml lysis buffer, incubated for 1 h, sonicated (Misonix sonicator (Misonix) with the following settings: micro tip, 30 s on, 2 min off, amplitude 70, 7 min total sonication time) and processed as described in Whyte *et al.*^31^ qPCR was carried out in the LightCycler480 (Roche) with a 15 min DNA denaturation step at 95 °C, followed by 50 cycles of 15 s at 95 °C, 30 s at 58 °C and 30 s at 72 °C. PCR measurements were performed in duplicate. Enrichments are expressed in percentage of input ((2−ΔCT)*100/(Vol IP/Vol input)). Averages and standard deviations of experimental replicates are shown in the figures.

### ChIP-Seq

10 ng, as quantified by Qubit dsDNA HS Assay Kit (Life Technologies), of input and of immuno-precipitated-material was used for library preparation. For one ChIP-Seq experiment chromatin prepared from 100 × 10^6^ HeLa-LTR-Luciferase cells was incubated with 10 μg of antibody (the same protocol was used for all antibodies except for antibodies against histone modifications for which chromatin of only 50 × 10^6^ HeLa-LTR-Luciferase cells was used). Under these conditions the following total amounts of DNA were precipitated:

An amount of 10 μg of anti-RNAPII total (Santa Cruz, sc-899): Replicate A: 53.7 ng. Replicate B: 71.1 ng.

An amount of 5 μg of anti-H3K4me3(Abcam, ab8580): Replicate A: 1290, ng. Replicate B: 1251, ng.

An amount of 10 μg of anti-NELF-E (Millipore, ABE48): Replicate A: 21 ng. Replicate B: 27 ng.

An amount of 10 μg of anti-Spt5 (BD Transduction Laboratories, 611106): Replicate A: 18 ng. Replicate B: 24 ng.10 μg of anti-INTS3 (Abcam, ab70451): Replicate A: 15 ng. Replicate B: 15.9 ng.

An amount of 10 μg of anti-INTS3 (Bethyl Laboratories, A302-051A): Replicate A: 81 ng. Replicate B: 81 ng.

An amount of 10 μg of anti-INTS3 (Proteintech, 16620-1-AP): Replicate A: 109 ng. Replicate B: 108.3 ng.

An amount of 10 μg of anti-INTS11 (Bethyl Laboratories, A301-274A): Replicate A: 10.8 ng. Replicate B: 13.2 ng. 10 μg of anti-INTS13 (Bethyl Laboratories, A303-575A): Replicate A: 33 ng. Replicate B: 43.5 ng. 10 μg of anti-INTS13 (Proteintech, 19892-1-AP): Replicate A: 45 ng. Replicate B: 55.2 ng.

Libraries were constructed using either Illumina ChIP-Seq DNA Sample Prep Kit (non-multiplexed libraries) or TruSeq ChIP Sample Prep Kit (multiplexed libraries) according to manufacturer’s instructions. Image analyses and base calling were performed using the HiSeq Control Software and Real-Time Analysis component (Illumina). Data quality was assessed using fastqc from the Babraham Institute and the software SAV (Sequence Analysis Viewer) (Illumina). De-multiplexing and alignment were performed using Illumina's sequencing analysis software (CASAVA 1.8.2) (Illumina).

### Luciferase assay

Luciferase activity was measured according to the manufacturer’s protocol (Promega). Luciferase activity was normalised to protein concentration using Bradford assay (Biorad).

### siRNA transfection

Knockdowns were performed using Interferin (Polyplus Transfection) as transfection reagent according to manufacturer’s instructions. HeLa-LTR-Luciferase cells were transfected for 5 h and then the medium was exchanged. In experiments in which HIV-LTR was activated, 24 h later cells were transduced with a retrovirus expressing Tat (Transactivating regulatory protein). NRO/ChIP/Luciferase assay experiments were performed 48 h after siRNA transfection.

For complementation experiment, INTS11 Knockdown was performed as described above using a siRNA directed against the 3′UTR of INTS11 mRNA (siINTS11-UTR). After 4 h of siRNA transfection the medium was exchanged and transfection of Flag-INTS11 plasmids was performed using jetPEI (Polyplus Transfection) as transfection reagent according to manufacturer’s instructions. Medium was exchanged again after 12 h and Luciferase assay experiments were performed 48 h after siRNA transfection.

### Nuclear run-on assays

NRO assays were performed as described previously using ~8 × 10^6^ nuclei prepared from HeLa-LTR-Luciferase cells^32^. NRO reactions were performed on 30 °C, stopped with Trizol LS (Life Technologies) and the RNA isolated following manufacturer’s instructions. RNAs transcribed during the assay were purified once on anti-BrdU antibody conjugated agarose beads (50 μl of a 25% slurry) and reverse transcribed using SuperScript III (Life Technologies) with random primers. In test experiments, NROs were performed either with Br-UTP or unlabelled UTP. The NRO protocol resulted in enrichment of labelled as compared with the unlabelled RNAs after one round of purification depending on the RNAs measured (Supplementary Fig. 3B). In test assays, enriched RNAs were measured as percentage of input. For knockdown experiments the average of two technical replicates was normalised to the four controls RPS14, 7SK, KDSR and PIGB either individually (see Supplementary Fig. 3C) or averaged (Fig. 2c) using the comparative CT method (2−ΔΔCT). Averages and standard deviations of 3 experimental replicates are shown in the figures. PCR measurements were performed in duplicate using SYBR Green (Qiagen). Amplification was carried out in the LightCycler480 (Roche) with a 15 min DNA denaturation step at 95 °C, followed by 50 cycles of: 15 s at 95 °C, 30 s at 58 °C and 30 s at 72 °C.

### Immunoprecipitations supplemented with RNAse or DNAse

Immunoprecipitations of eNELF-E treated with RNAse or DNAse were performed as in section ‘Immuno-purification of NELF-E’ except that before incubation with anti-FLAG antibody-conjugated agarose beads the nuclear extracts were treated with 0.1 mg ml^−1^ EtBr (Sigma-Aldrich) and 0.4 U μl^−1^ DNaseI (Sigma-Aldrich) for 20 min at room temperature to disrupt protein/DNA interactions. Similarly, to disrupt protein/RNA interactions nuclear extracts were treated with 10 μl 1 ml^−1^ RNAse Cocktail (RNase A at 500 U ml^−1^;RNaseT1 at 20,000 U ml^−1^, Life Technologies) and 10 μl 1 ml^−1^ RNAse A (24 mg ml^−1^, Sigma-Aldrich) for 20 min at room temperature before incubation with anti-FLAG beads. RNAse treated immunoprecipitations of CyclinT1 and Hexim were done accordingly using primary antibodies specified in the list of primary antibodies.

### Monitoring the cell cycle

To label DNA synthesis in different siRNA backgrounds the Click-iT Edu Flow Cytometry Assay Kit (Life Technologies) was used with a pulse of EdU (10 μM) for 1 h following manufacturer’s instructions. Staining of DNA was performed using Fx Cycle (Life Technologies). Cell cycle distributions of KD cells were acquired by flow cytometry.

### Whole RNA extraction and quantification

RNA was isolated from ~1 × 10^6^ HeLa-LTR-Luciferase cells with Trizol (Life Technologies). Contaminating DNA was digested by TURBO DNAse (Life Technologies) prior to the reverse transcription reaction primed either with random or oligo dT primers using SuperScript III (Life Technologies). PCR measurements were performed in duplicate using SYBR Green (Qiagen). Amplification was carried out in the LightCycler480 (Roche) with a 15 min DNA denaturation step at 95 °C, followed by 50 cycles of: 15 s at 95 °C, 30 s at 58 °C and 30 s at 72 °C. The average of the technical replicates was normalised to RPS14 (see list of primers) levels using the comparative CT method (2−ΔΔCT). Averages and standard deviations of three experiments are shown in the figures.

### RNASeq

RNASeq libraries were constructed using the TruSeq stranded mRNA sample preparation kit from Illumina. cDNA libraries of polyadenylated RNAs were generated using 1 μg of whole-cell RNAs following Illumina’s instructions. For cDNA libraries of total RNAs whole-cell RNAs were depleted for ribosomal RNAs using the Ribo-Zero Magnetic Gold Kit following manufacturer’s instructions. 100 ng of ribosomal RNA depleted whole-cell RNA was used for library construction following Illumina’s instructions. Image analysis and base calling were performed using the HiSeq Control Software and Real-Time Analysis component. Data quality was assessed using fastqc from the Babraham Institute and the Illumina software SAV (Sequence Analysis Viewer). De-multiplexing was performed using Illumina's sequencing analysis software (CASAVA 1.8.2).

### Microarrays

For the preparation of Cy3- and Cy5- labelled aRNA 1 microgram of total RNA/sample was amplified and labelled using the Amino Allyl Message Amp II aRNA Amplification Kit (Ambion; Austin, TX, USA), according to manufacturer’s instructions. Labelled aRNAs were added to Hybridisation Buffer, hybridisation component A and alignment oligo (Roche Nimblegen), denaturated at 95 °C for three minutes and applied to an array of a 12 × 135 K Nimblegen HG18_100718 microarray slide. Hybridisation was carried out at 42 °C for 16 h in hybridisation system 4 (Roche Nimblegen). Hybridised slides were washed according to Nimblegen's protocol. Microarrays were scanned at 1 μm resolution in both Cy3 and Cy5 channels with Innoscan900 scanner (Innopsys, Carbonne, France) with variable photo multiplier tube settings to obtain maximal signal intensities. Nimblescan v2.5 software (Roche Nimblegen) was used for feature extraction. Data was stored and visualised using the BASE data management software.

Differential expression (DE) was analysed in R using limma (Smyth GK, PMID: 16646809). Genes were considered to be DE if BH adjusted *P*-value was below 0.01 ('adj.P.Val', see column 14 of Supplementary Dataset 2B–D).

### Statistical analyses of ChIP-Seq data

ChIP-Seq peaks of NELF, INTS3 and INTS11 were identified using MACS2 with normalisation to the corresponding input sequenced in parallel. Peaks within promoter regions were then intersected and enrichment tested statistically using Fisher exact test. ChIP-Seq data for RNAPII upon various depletions were further analysed using a rMAT package by counting normalised (r.p.m.) ChIP-Seq reads in the indicated windows with respect to TSS (−250 to +250; +500 to +1000, +500 to the end of genes or from −500 to 0 of termination sites). RNAPII pausing indices were calculated as previously^10^ as a ratio of normalised ChIP-Seq reads on TSSs (+/− 250 bp) over that of the corresponding bodies (+500 to +1000 from TSSs). The RNAPII processivity index was measured as the log ration of normalised ChIP-Seq reads of RNAPII in gene bodies over termination sites (−500 to 0 from TES). To estimate changes in processivity, pair-wise wilcoxon tests were performed between the same set of genes, in two conditions (for example, INTS11-KD and control). Proportional Venn diagrams were plotted using http://bioinforx.com. Statistical analyses of ChIP-Seq by principal component analysis (PCA) were performed using the package FactoMiner from R by taking the total (normalised) read counts as measured from our ChIP-Seq data within windows corresponding to the indicated regions. The generated averaged values were then provided for each ChIP-Seq and for each individual gene allowing to perform clustering ascendant hierarchical for all centred and normalised data using the package FactoMiner from R. Clustering ascendant hierarchical measures Ward distances reflecting the minimal variance among all data sets provided^33^, which was performed separately for genes harbouring NELF, Spt5, INTS11 and INTS3 or not. For comparison of data among different sets of genes (for example, Fig. 3h according to binding of NELF/INTS11/INTS3), a simple Wilcoxon test was used. For comparison of mock-depleted (scrambled) control with INTS3- or INTS11-depleted samples, a pair-wise Wilcoxon test was performed between each condition and for every group of genes defined according to the presence or not of NELF, INTS3 and/or INTS11 peaks as indicated. Motif scan was performed by systematically recording of the motifs found in each group of ChIP-Seq peaks (INTS3/11 and NELF) using RSAT-tools^34^ ( www.rsat.ulb.ac.be/rsat/) and the JASPAR database. The relevance of the identified motifs was then tested for enrichment by intersection analysis with a list of INTS11 and/or NELF ChIP-Seq peaks through fisher exact test (see Supplementary Fig. 6a). Only the motifs with a significant overlap with ChIP-Seq peaks (*P*<1e-3) were further analysed for their enrichment in up- or down-regulated genes (Fig. 4) or in genes harbouring processivity defects upon INTS11-KD (Fig. 5). A complete list of all motifs is provided in Supplementary Dataset 5. Fisher exact tests were performed to test which of these motifs (versus no motif) was specifically enriched among the indicated group of up-/down-regulated genes upon INTS11-KD and/or NELF-KD, and for RNASeq −/+ reads.

### Statistical analyses of gene expression data

Gene expression analyses were performed through both microarrays and strand-specific RNASeq analyses from three independent pools of cells efficiently depleted of INTS3, INTS11 or NELF as compared with mock-depleted control cells. Briefly, following the alignment of sequence reads on the human genome version hg19 with TopHat 2.0.9, all subsequent analyses were done in R 3.0.2 ( www.r-project.org) with Bioconductor packages ( www.bioconductor.org), including GenomicRanges 1.14.4 (ref. 35), Rsamtools 1.14.3 and Homo.sapiens 1.1.2. Coverage data for each gene (exons only) was calculated from aligned RNASeq reads. For each sample, the processivity index was calculated as the ratio of coverage for the (TES-500 bp, TES) region to the (TSS+500 bp, TSS+1000, bp) region. Differential processivity in total and polyA+ RNASeq data was assessed using a moderated Student's t-statistic as implemented in the limma Bioconductor package^36^. Raw *P*-values were adjusted for multiple testing using the *q*-value package^37^. Genes with various *q*-value<1, 5 or 20% were used to test the reproducibility of the measure of differential processivity from triplicates and then between INTS11-KD and control samples.

For heat maps, ChIP-Seq peaks identified using MACS2 were intersected with all TSSs followed by statistical test for enrichment in DE gene lists (Fisher exact test; Fig. 3). Log *P*-values were obtained after applying the Hochberg & Benjamini corrections. RNASeq analyses (RNASeq; Hi-Seq2500; Illumina) were performed from three independent replicates for each condition. Alignments were performed using the Burrows–Wheeler alignment tool software on genome annotations of release H19 of human database for parsing and the EdgeR package for identification of the DE genes (up- or down-regulated; *P*-value<0.001) from the three independent replicates. The specificity of the lists of DE genes was functionally verified through other packages (for example, DESeq2) of through manual read counting with HTseq. Proportional Venn diagrams were plotted using http://bioinforx.com. Statistical enrichments according to the presence or not of ChIP-Seq peaks of INTS11, INTS3, and NELF were performed by intersecting groups of genes using fisher's exact tests. *P*-values were corrected using the Benjamini & Hochberg multiple testing corrections. For specificity of the RNASeq reads, ‘lone’ genes were pre-selected to eliminate any background RNASeq signal from overlapping genes (for example, divergently transcribing genes). RNASeq reads were normalised (RPKM) for all conditions and variations were scored as a Log ratio between control and depleted (INTS11-KD) conditions. RNA processivity index were measured for total RNAs (ribo-depleted RNAs) or polyA+ RNAs as the log ratio of normalised RNASeq reads in gene bodies (+500 to+1000 from TSSs) over the corresponding termination site (−500 to 0 from TES). For statistical tests, QQ-plots were used to validate the normal distribution of the variations in processivity providing with confidential intervals using the ‘pnorm’ function of R as the probability of obtaining the change in processivity. Pair-wise wilcoxon test was then used to statistically test whether variations in processivity were relevant for a given subset of genes. In this case, we compared such variations to that obtained for a group of genes that were active (similar expression levels according to RNASeq reads) yet that showed were not deregulated upon INTS11-KD and NELF-KD. We also used ‘unbound’ genes (genes with no ChIP-Seq peak for NELF, INTS11 or INTS3) as an additional control. Variations in strand-specific RNASeq+/− reads were analysed separately by comparison between INT11-KD and control/mock-depleted cells. Significant variations in RNASeq- reads were scored using non-overlapping, ‘lone genes’ (every gene with no overlapping genes in the ±5 Kbp surrounding window). Variations in read counts were scored within the −2000 to 0 windows from their TSSs from three independent replicates and normalised to the total number of reads in the window. In Fig. 4e, control genes with no significant change in expression (grey boxes) were scored by DESeq2 as genes harbouring similar levels of RNASeq reads in INTS11-KD and NELF- KD as compared to control cells.

## Author contributions

M.B., B.S. and O.C. conceived the study and wrote the paper. M.B., B.S. and O.C. designed experiments and interpreted data. B.S., N.M. and S.B. performed experiments. O.C., A.G., G.M., P.G.M., R.R. and S.K. performed the bioinformatics analyses. All the authors discussed the data.

## Additional information

**Accession codes:** All ChIP-Seq and RNASeq data are available at the NCBI's GEO database under the accession number GSE60586 for SuperSeries including GSE60584 and GSE60585 subseries.

**How to cite this article:** Stadelmayer, B. *et al.* Integrator complex regulates NELF-mediated RNA polymerase II pause/release and processivity at coding genes. *Nat. Commun.* 5:5531 doi: 10.1038/ncomms6531 (2014).

## Supplementary Material

Supplementary Figures, Methods & References Supplementary Figures 1-16, Supplementary Methods and Supplementary References

Supplementary Dataset 1 Number of unique peptides recovered for eNELF-E associated factors identified by tandem mass spectrometry.

Supplementary Dataset 2 List of up- or down- regulated genes as obtained from microarray analysis in INTS11-KD, INTS3-KD, NELF-KD compared to scrambled control in triplicates (see Methods). List of differentially expressed genes as obtained by limma analysis of microarray data upon NELF-KD (B), INTS3-KD (C), INTS11-KD (D) or scrambled control performed from three independent experiments (see Supplementary Methods).

Supplementary Dataset 3 List of genes harboring a ChIP-Seq peak for INTS11, INTS3, NELF, Spt5 as obtained from MACS2 (see Methods).

Supplementary Dataset 4 List of differentially expressed genes as obtained from RNASeq analysis in INTS11-KD, INTS3-KD, NELF-KD after purification of polyA+ RNAs or not (for 'total' RNAs; see Methods).

Supplementary Dataset 5 List of genes harboring the indicated DNA consensus motifs as identified through systematic search of motifs in all ChIP-Seq peaks of INTS11/3 or NELF. ChIP-Seq peaks were attributed to genes based on localization surrounding TSSs (+/- 250 bp from TSS) or termination site (3' box; -1000 to 0 from termination site/TES).

Supplementary Dataset 6 List of genes harboring RNA processivity defects upon INTS11- or NELF- depletion (dataset 6A and dataset 6B respectively) for total RNAs (ribo-depleted RNAs) or polyA+ RNAs (see Methods). Processivity was calculated in each condition as a log ratio of normalized RNASeq reads in gene bodies (+500 to + 1000 from TSSs) normalized to the corresponding termination site (-500 to 0 from TES; see Methods).
