# Supplementary material for: Integrator complex regulates NELF-mediated RNA polymerase II pause/release and processivity at coding genes
Source: Nat Commun. 2014 Nov 20;5:5531. doi: 10.1038/ncomms6531 (PMC4263189; doi:10.1038/ncomms6531)
Supplement: Supplementary Figures, Methods & References — Supplementary Figures 1-16, Supplementary Methods and Supplementary References [file ncomms6531-s1.pdf]

Supplementary figures

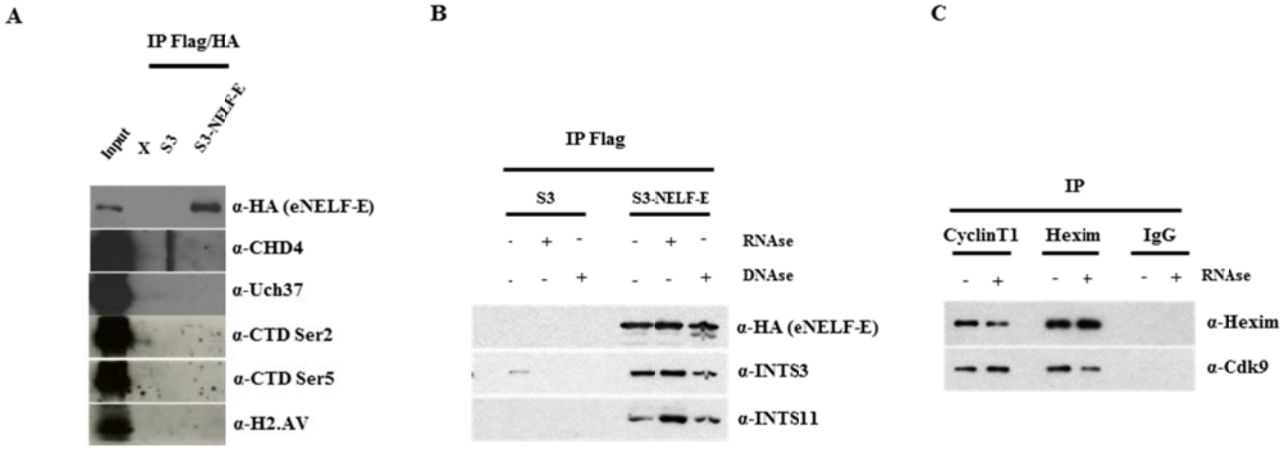

Supplementary figure 1

A. Flag/HA IP from samples used in Figure 1B were separated by SDS-PAGE and the presence of eNELF-E-associated proteins identified was confirmed by immuno-blotting. eNELF-E does not bind CHD4, Uch37, H2.AV, RNAPII phosphorylated on Ser2 or RNAPII phosphorylated on Ser5. Interaction between NELF and INTScom is DNA and RNA independent.

B. Immunoprecipitations of eNELF-E with RNAse and DNAse treatment were performed as described in Figure 1 except that before incubation with anti-FLAG antibody-conjugated agarose beads the nuclear extracts were supplemented with either 0.1 mg/ml EtBr (Sigma-Aldrich) and 0.4 U/µL DNaseI (Sigma-Aldrich) or 10µl/1ml RNAse Cocktail (RNase A at 500U/mL;RNaseT1 at 20,000U/mL, Life Technologies) and 10µl/1ml RNAse A (24mg/ml, Sigma) for 20min at room temperature to disrupt protein/DNA or protein/RNA interactions, respectively.

C. RNAse treatment was controlled by monitoring the RNA dependent interaction between pTEFb (CyclinT1/Cdk9) and Hexim. Accordingly, before anti-cyclinT1, anti-Hexim1 or unspecific IgG IPs nuclear extracts were treated with RNAse as described in B.

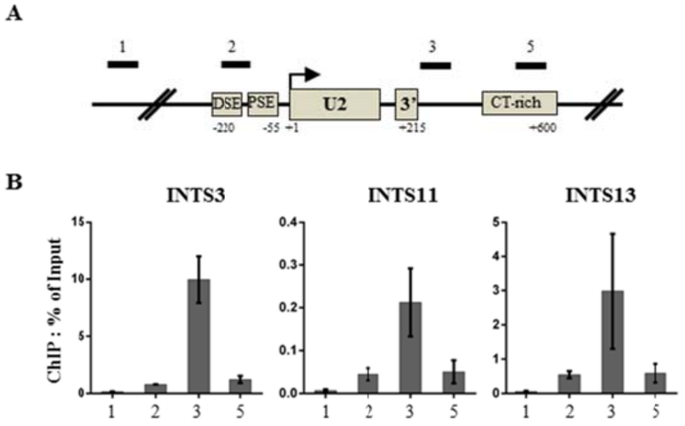

Supplementary figure 2

A. Schematic representation of snRNA U2 locus indicating positions of primers used in ChIP assays. Primers used were previously published[1].

B. Antibodies anti-INTS3 (Bethyl Laboratories), anti-INTS11 (Bethyl Laboratories) and anti-INTS13 (Protein Tech) used for ChIP experiments on HIV-LTR reproduce ChIP profiles published in[1], peaking at amplicon 3.

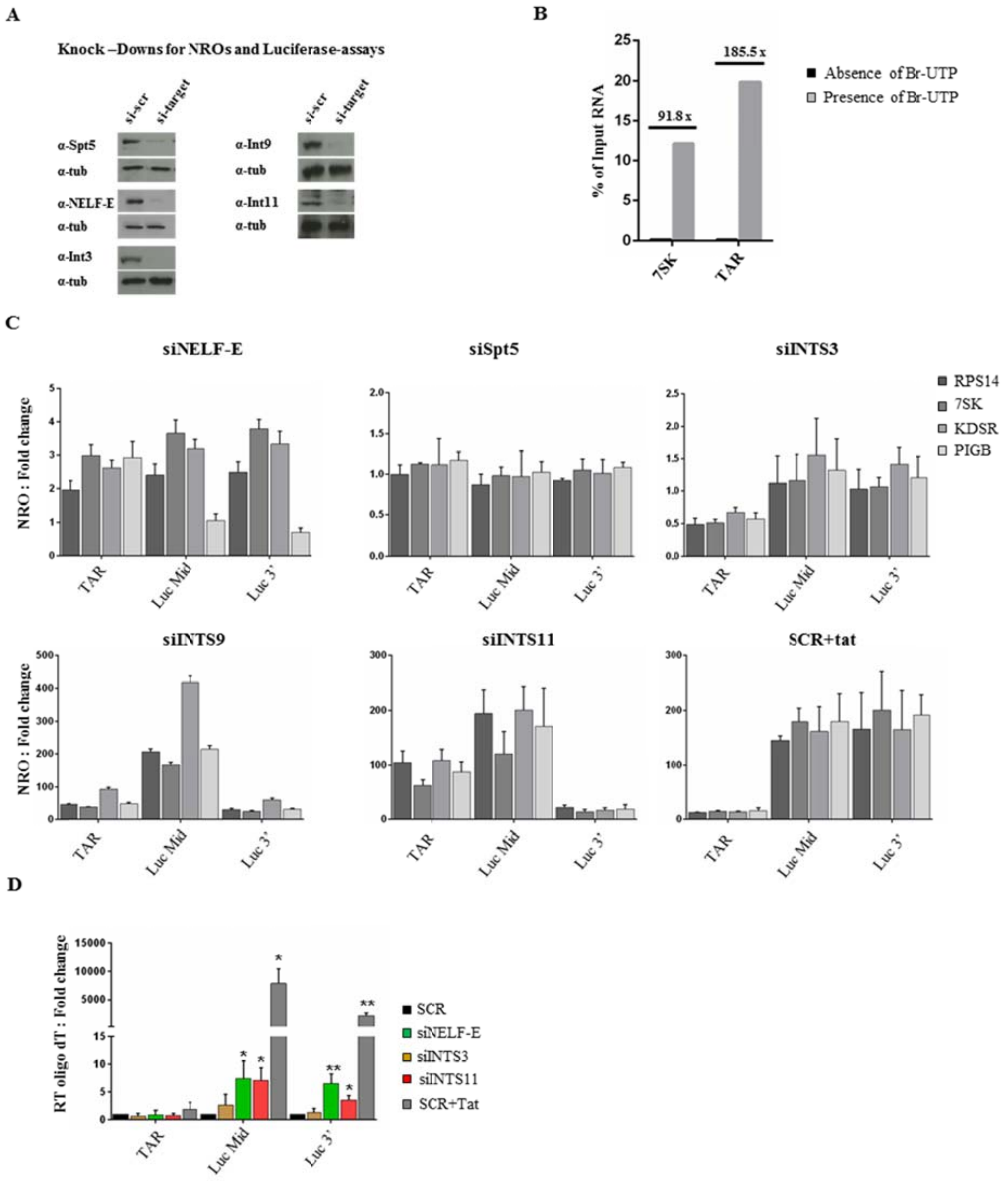

Supplementary figure 3

A. Knockdown efficiencies of siSpt5-, siNELF-E- and siINTS3/9/11-siRNAs as compared to control siRNA (si-scr) for NRO and luciferase experiments. Tubulin was used as loading control.

B. Enrichment of labeled (Br-UTP) RNAs synthesized during NRO assays. NRO reactions were performed either in the presence or absence of labeled Br-UTP, in the absence of Br-UTP the NRO reactions were performed with unmodified UTP. Labeled RNAs transcribed during the assay were purified once using anti-BrdU antibody conjugated agarose beads. For the 2 conditions purification efficacy of labeled RNA was measured as percentage of input RNA. Enrichments of labeled as compared to non-labeled RNAs are shown for 7SK and TAR.

C. Normalizations of NROs by different control genes are concordant. For NRO experiments the average of 2 technical replicates was normalized to the 4 controls RPS14, 7SK, KDSR and PIGB. For every siRNA knock-down the 4 different normalized NRO profiles are plotted.

D. Whole RNA was extracted from HeLa-LTR-Luc cells transfected with the indicated siRNAs. Reverse transcription was primed with oligo dT primers. Values were normalized to the amount of RPS14 RNA in the same samples. Results are presented as fold change over control condition SCR. *= p-value < 0.05; **= p-value < 0.005; ***= p-value < 0.0005, no*=no significant p-value as measured by student's t-test. Error bars represent standard deviations.

A

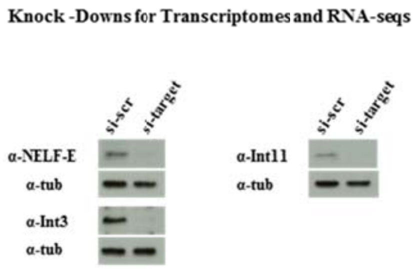

B

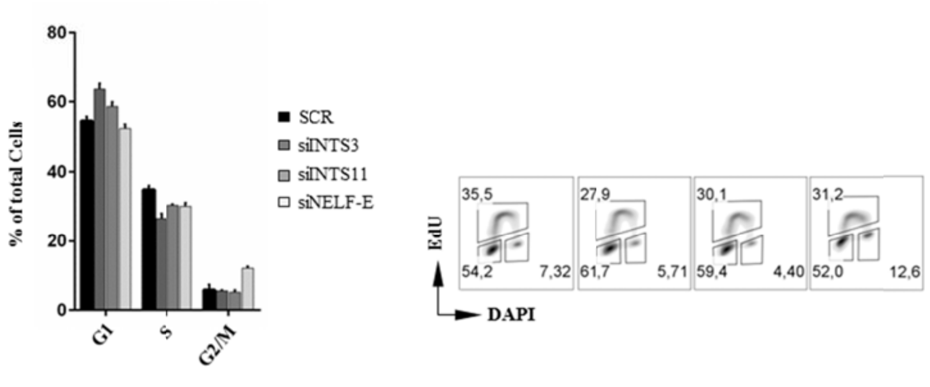

Supplementary figure 4

A. KD efficiencies of siNELF-E and siINTS3/11-siRNAs as compared to siRNA siscr for transcriptomic and RNA-seq experiments. Tubulin was used as loading control.

B. Cell cycle status was evaluated by Flow cytometry after 48h of siRNA treatment by measuring incorporation of EdU (1h pulse) and nuclear content (DAPI). Scatter plots are from one representative experiment (n = 3).

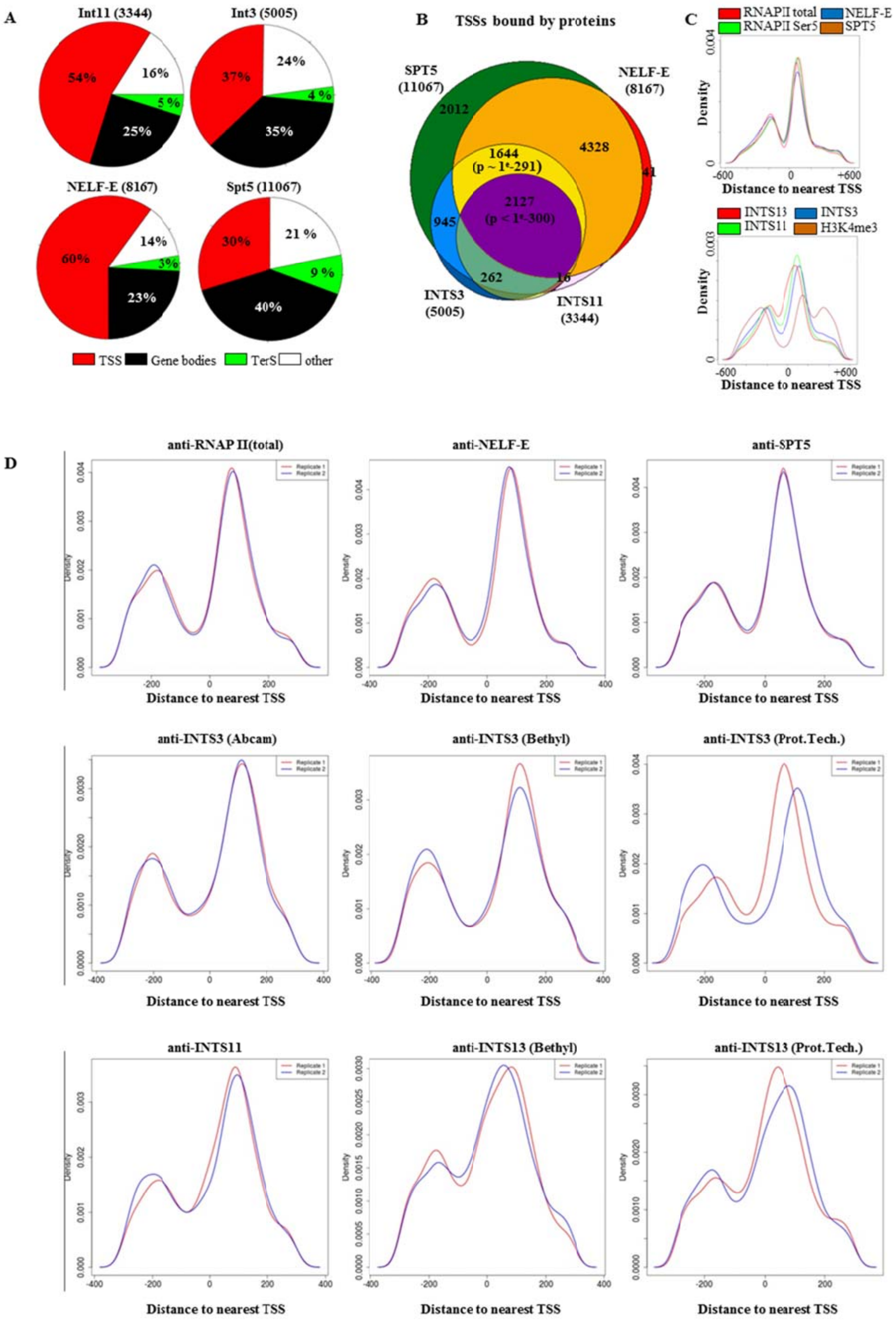
A
Int11 (3344)
Int3 (5005)
NELF-E (8167)
Spt5 (11067)
54%
16%
5 %
25%
37%
24%
4 %
35%
60%
14%
3%
23%
30%
21 %
9 %
40%
TSS
Gene bodies
TerS
other
B
TSSs bound by proteins
SPT5 (11067)
NELF-E (8167)
2012
4328
41
1644 (p ~ 1e-291)
2127 (p < 1e-300)
945
262
16
INTS3 (5005)
INTS11 (3344)
C
RNAPII total
NELF-E
RNAPII Ser5
SPT5
Density
Distance to nearest TSS
INTS13
INTS3
INTS11
H3K4me3
D
anti-RNAP II(total)
anti-NELF-E
anti-SPT5
anti-INTS3 (Abcam)
anti-INTS3 (Bethyl)
anti-INTS3 (Prot.Tech.)
anti-INTS11
anti-INTS13 (Bethyl)
anti-INTS13 (Prot.Tech.)
Distance to nearest TSS

Supplementary figure 5

A. Genome-wide distribution of ChIP-Seq peaks of INTS3, INTS11, NELF-E and SPT5 with respect to the TSS, bodies or termination sites of transcription (‘terS’) of cellular, coding genes versus other genomic regions (‘other’).

B. Venn diagram showing the intersection analysis of NELF-E-, Spt5- INTS3- and INTS11-binding sites as scored by ChIP-Seq using MACS2 for peak detection. The p-value of quadruple intersection is p~ 1e-300 as tested by Fisher exact test. Note that the intersection of NELF/Spt5 unique to INTS3 (without INTS11) is also significant (1644 genes; p~ 1e-291) by contrast to any combination of INTS11 with other factor in the absence of INTS3 (16 genes).

C. Density plots representing the averaged distribution of the summits of ChIP-Seq peaks with respect to TSSs (x-axis, position ‘0’) for NELF, Spt5, INTS3/11/13, for RNAPII phosphorylated at Ser5 or total RNAPII and for the histone H3 tri-methylated on lysine 4 (H3K4me3).

D. Reproducibility of ChIP-seq experiments in biological replicates as visualized by plotting distances of ChIP-Seq peak summits to TSSs.

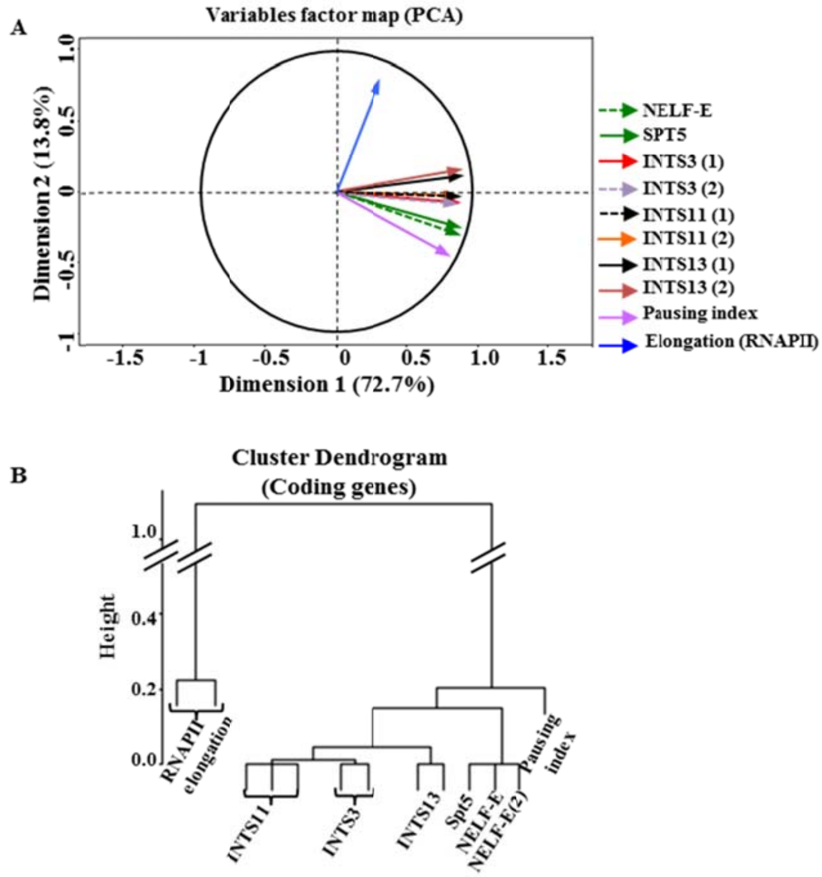

Supplementary figure 6

A. Variables factor map showing the overall correlation in binding of the indicated factors using normalized ChIP-Seq data (see Supplementary Methods), as obtained by running Principal component analysis (PCA)[2]. The map shows the tight correlation among the variations in ChIP-Seq reads of NELF, SPT5, INTS3, INTS11, INTS13 or RNAPII as quantified surrounding the TSS (+/- 250 bp) for all genes The values for RNAPII pausing were calculated as previously[3] as the ratio of ChIP-Seq reads of RNAPII over the TSSs (+/- 250bp) over ChIP-Seq reads of RNAPII over gene bodies (+500 to +1000). For RNAPII ‘elongation’, values were obtained by quantifying ChIP-Seq reads over gene bodies (+500 to +1000). Numbers (e.g. “INTS3 (1)” or “INTS3 (2)”) correspond to quantification of ChIP-Seq reads as obtained from independent ChIP-Seq replicates. Note that the x- and y-axis of the map represent the two most representative dimensions of the variations in ChIP-Seq reads among all data set[2].

B. Dendrogram representing the genome-wide correlations among the indicated ChIP-Seq data after running Clustering Ascendant Hierarchical (CAH) with Ward distance[2] to measure the minimal variance for all data set. The height (y-axis) reflects the co-variance among ChIP-Seq data.

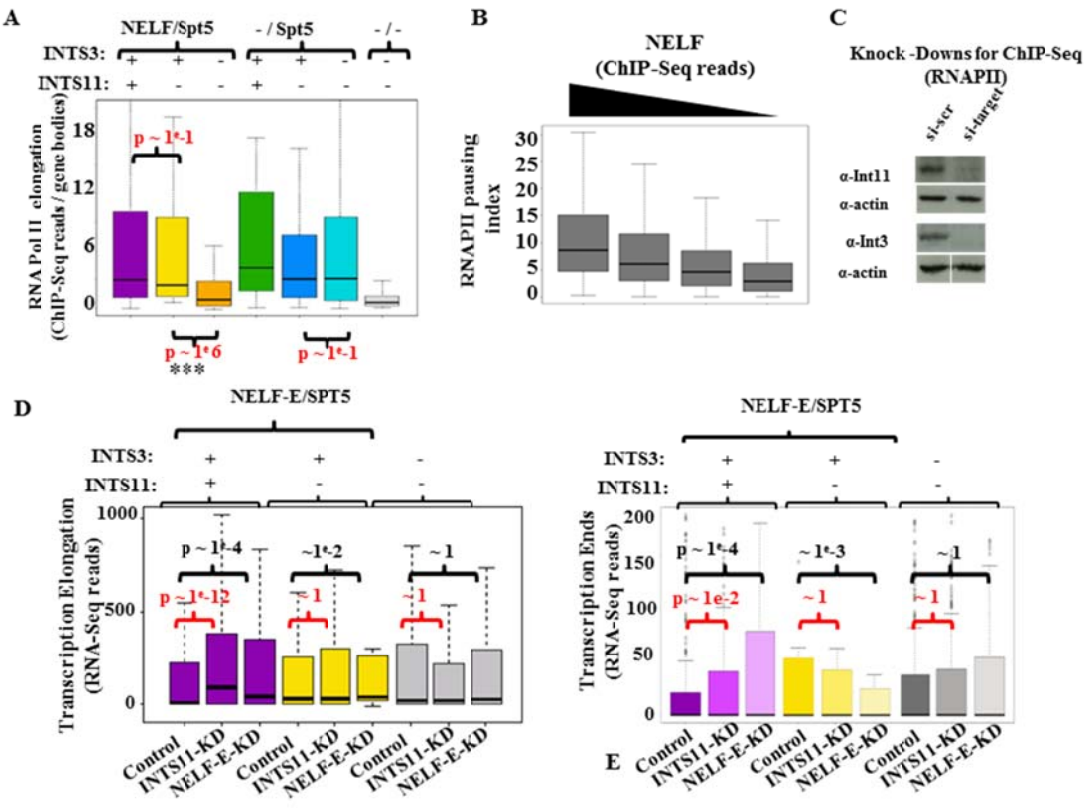

Supplementary figure 7

A. Box plot representing the measure of RNAPII elongation as a function of the binding or not of NELF together with INTS3 and/or INTS11 in promoter regions (+/- 250 bp around TSSs). See also Fig.3f for a similar correlation between NELF/INTS3/11 binding and RNAPII pausing index.

B. Box plot representing the measure of RNAPII pausing calculated as previously [3] as a function of NELF binding to promoter regions (+/- 250 bp around TSSs). To discriminate the specific correlation of NELF binding with RNAPII pausing over its overall correlation with RNAPII occupancy, correlations were calculated by among the top 10,000 genes with higher RNAPII levels. Genes were ranked in 4 quartiles (2,500 genes each) and RNAPII pausing index was then measured for each gene as previously reported [3].

C. Knockdown efficiencies of siINTS3/11-siRNAs as compared to siRNA siscr for RNAPII-ChIP-seq experiments. Actin was used as loading control.

D. Box plots showing the variations in RNASeq reads (total RNA) between control and INTS11-KD cells over the first exons (left box plot) or the transcription ends (right box plot), for 'direct targets' (genes that are bound by NELF-E/INTS3/INTS11; for a total of 859 direct targets). The indicated p-values correspond to pair wise wilcoxon tests between control and INTS11-depleted samples.

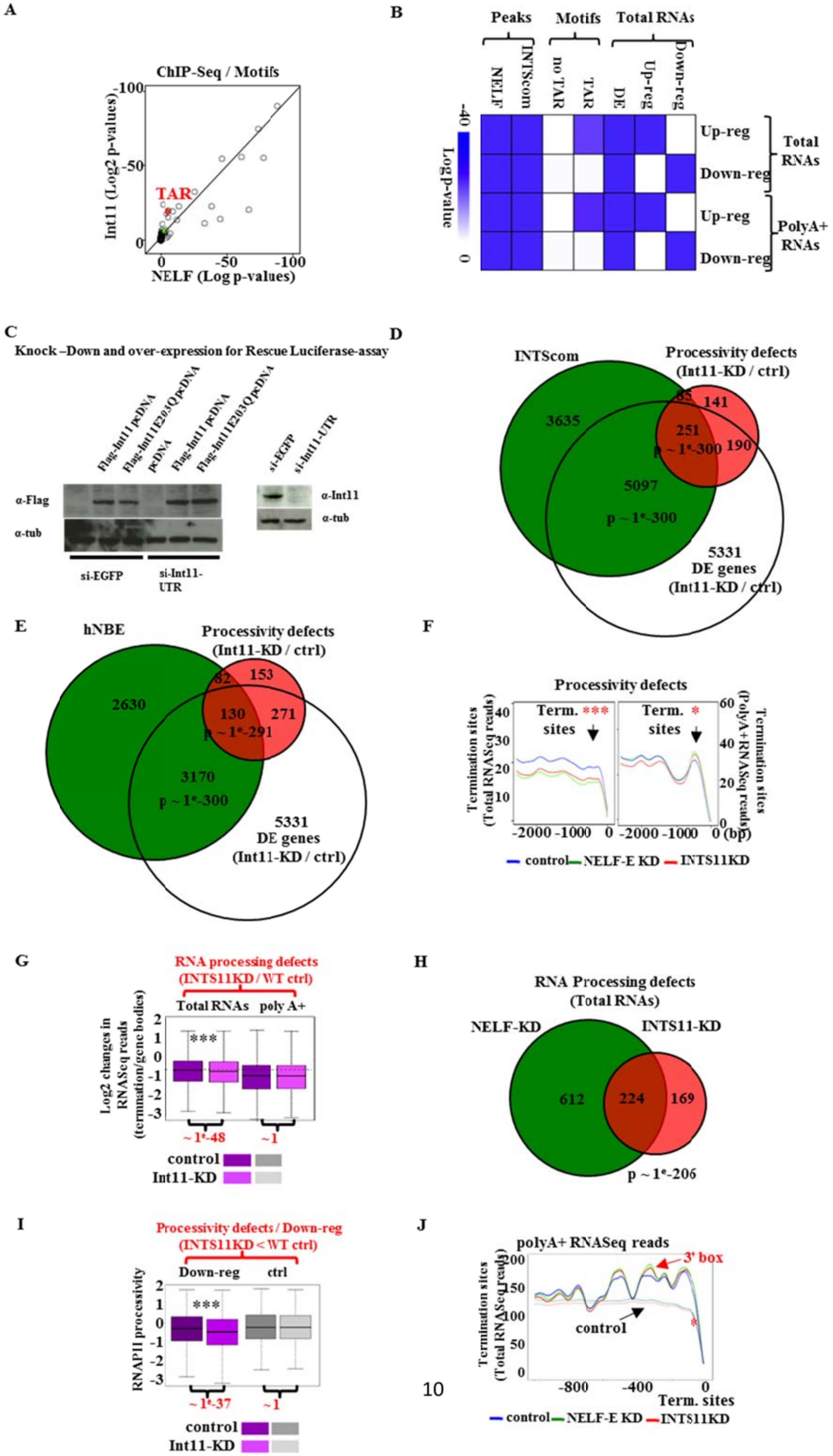
A
ChIP-Seq / Motifs
Int11 (Log2 p-values)
NELF (Log p-values)
TAR
B
Peaks
Motifs
Total RNAs
NELF
INTScom
no TAR
TAR
DE
Up-reg
Down-reg
Log p-value
-40
0
Up-reg
Down-reg
Total RNAs
PolyA+ RNAs
C
Knock –Down and over-expression for Rescue Luciferase-assay
Flag-Int11 pcDNA
Flag-Int11E203Q pcDNA
pcDNA
si-EGFP
si-Int11-UTR
α-Flag
α-tub
α-Int11
D
INTScom
Processivity defects (Int11-KD / ctrl)
3635
85
141
251
p ~ 1e-300
190
5097
p ~ 1e-300
5331 DE genes (Int11-KD / ctrl)
E
hNBE
Processivity defects (Int11-KD / ctrl)
2630
82
153
130
271
p ~ 1e-291
3170
p ~ 1e-300
5331 DE genes (Int11-KD / ctrl)
F
Processivity defects
Termination sites (Total RNASeq reads)
Termination sites (PolyA+RNASeq reads)
Term. sites ***
Term. sites *
(bp)
control
NELF-E KD
INTS11KD
G
RNA processing defects (INTS11KD / WT ctrl)
Total RNAs
poly A+
Log2 changes in RNASeq reads (termnation/gene bodies)
***
~ 1e-48
~ 1
control
Int11-KD
H
RNA Processing defects (Total RNAs)
NELF-KD
INTS11-KD
612
224
169
p ~ 1e-206
I
Processivity defects / Down-reg (INTS11KD < WT ctrl)
Down-reg
ctrl
RNAPII processivity
***
~ 1e-37
~ 1
control
Int11-KD
J
polyA+ RNASeq reads
3' box
Termination sites (Total RNASeq reads)
control
Term. sites
control
NELF-E KD
INTS11KD

Supplementary figure 8
A. Scatter plot showing the log p-values (as obtained by fisher exact tests) corresponding to intersections between the lists of genes that harbor one of the consensus motifs identified by systematic search of motifs near (+/- 100 bp) surrounding the center of ChIP-Seq peaks of INTS11/3/ or NELF -with the actual number of genes harboring a peak within their TSS together or not with that motif (see Methods). The red dot marks the 'TAR' motif (CUGGGA consensus). Only motifs showing significant intersections (fisher exact test < 1e-3) were further analyzed (see text).
B. Intersection analysis (in Log p-values; as obtained by fisher exact test) between the lists of genes that harbor one of the consensus motifs (see panel A) with genes that were up- or down- regulated upon INTS11-KD and that were bound by NELF and/or INTS subunits.
C. Western blotting analysis to verify the depletion/rescue of Flag- INTS11 catalytic mutant (or WT) corresponding to the experiment shown in Fig. 5D.
D. Venn diagram showing the statistical enrichment (in Log p-values; as obtained by fisher exact test) by triple intersection analysis among INTScom binding (INTS11 and INTS3), group of genes with RNA processivity defects (or 'termination defects', scored as significant variations of processivity between INTS11-KD compared to control cells that were uniquely found for total RNAs but not polyA+ RNAs). DE genes, differentially expressed genes (as obtained by DESeq2; see Methods)
E. Same as in panel C except that the group of genes harboring a TAR motif near their TSSs was considered instead of INTScom binding.
F. Averaged RNA+ profiles (y axis; normalized (RPM) RNASeq+ read counts) for genes with significant RNA processivity defects upon INTS11-KD as compared to control cells and that were uniquely detected when scoring such defects from RNASeq performed with total RNAs but not polyA+ RNAs. The averaged RNA+ profiles are also shown for total and polyA+ RNAs isolated from cells depleted of NELF.
G. Box plot showing the RNA processing index in INTS11-KD as compared to control cells (RNASeq reads over termination sites/reads in gene bodies) for the same set of genes corresponding to panel E. The purple color of the box represents the enrichment of such genes harboring RNA processing defects upon INTS11-KD in the lists of genes that are bound by INTScom and NELF (see Supplementary dataset 6).
H. Venn diagram showing the intersection analysis between genes harboring RNA processing defects in INTS11-KD and those harboring RNA processing defects in NELF- KD (as scored for genes with RNA processing defects uniquely found with total RNAs (but not polyA+ RNAs).
I. Box plot showing the RNAPII processivity index in INTS11-KD as compared to control cells for the group of down-regulated genes upon INTS11-KD (as obtained by DESeq2; see Methods). As a control set of genes (grey boxes), we took a group of genes with similar expression levels (as determined by RNASeq read counts) yet with no significant changes in expression (see Methods).
J. RNASeq+ profiles corresponding to RNASeq performed after polyA+ selection in INTS11-KD, NELF-KD control cells (see also Fig. 5F for the corresponding RNASeq+ profile obtained without polyA+ selection) and for genes bound by INTScom subunits in the presence or not of a 3' box near termination sites (-1000 to – from TES).

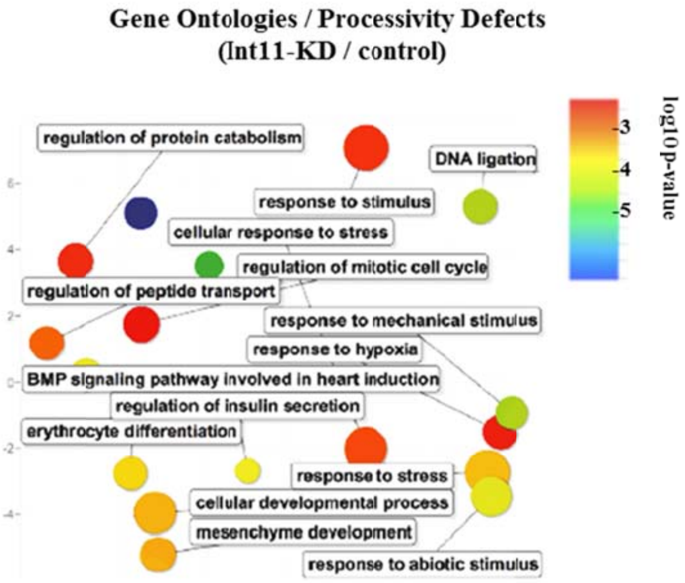

Supplementary figure 9

A. Gene ontologies associated with RNA processivity defects upon INTS11-KD as compared to control cells.

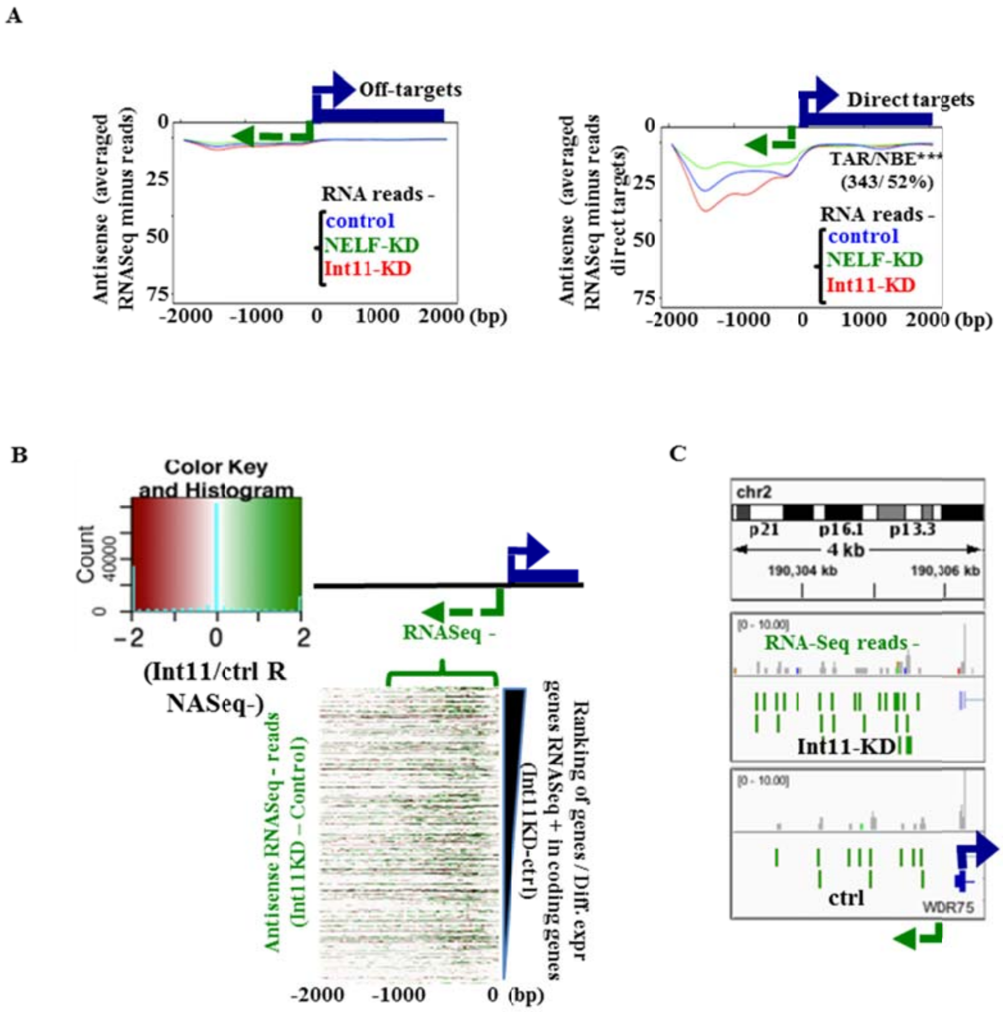

Supplementary figure 10

A. Averaged levels of strand-specific, RNASeq '-' reads in the upstream regions flanking control (left plot) or deregulated genes (upon INTS11-KD, as scored by DESeq2; right plot) in control, INTS11-KD or NELF-KD cells (blue, red and green curve, respectively). Note that > 52% of TSSs harboring the TAR/hNBE consensus are found among genes with higher levels of antisense reads upon INTS11-KD (fisher exact test p~ 1e-212; see text).

B. Heat map showing the net increase in strand-specific, RNASeq '-' reads between INTS11-KD and control cells (fold changes in reads in INTS11-KD compared to reads in control) in the upstream region flanking coding genes, for genes ranked according to differential expression levels (RNASeq+ reads) between INTS11-KD and control cells (top: most differentially expressed genes).

C. Example of deregulated gene upon INTS11-KD compared to control cells where increasing levels of RNASeq '-' reads are often detected in the upstream region (upon INTS11-KD/ control cells).

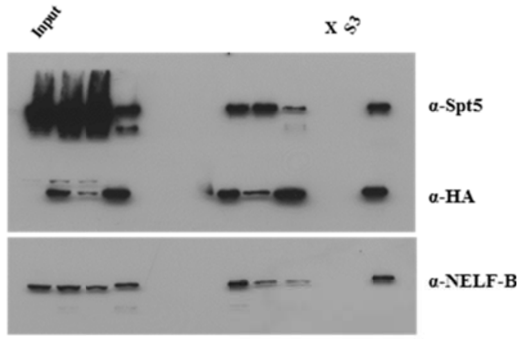

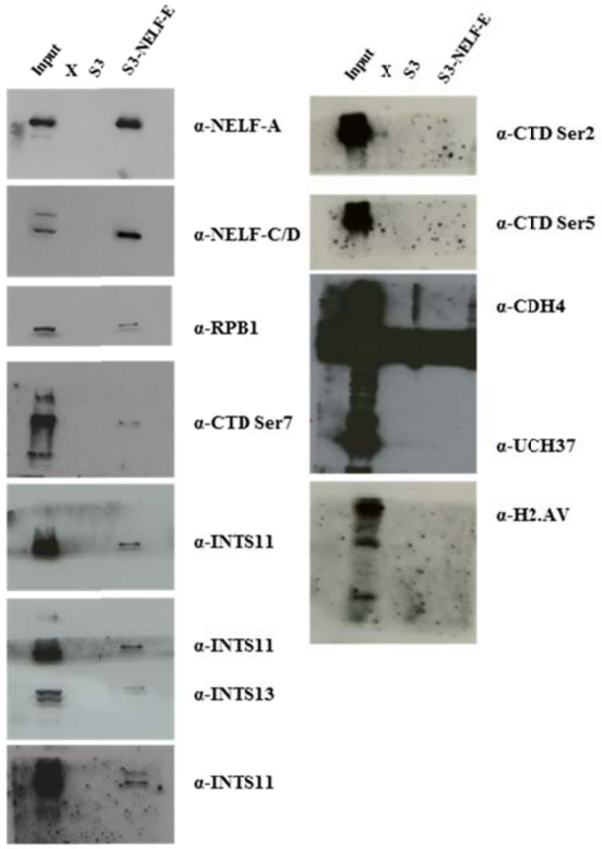

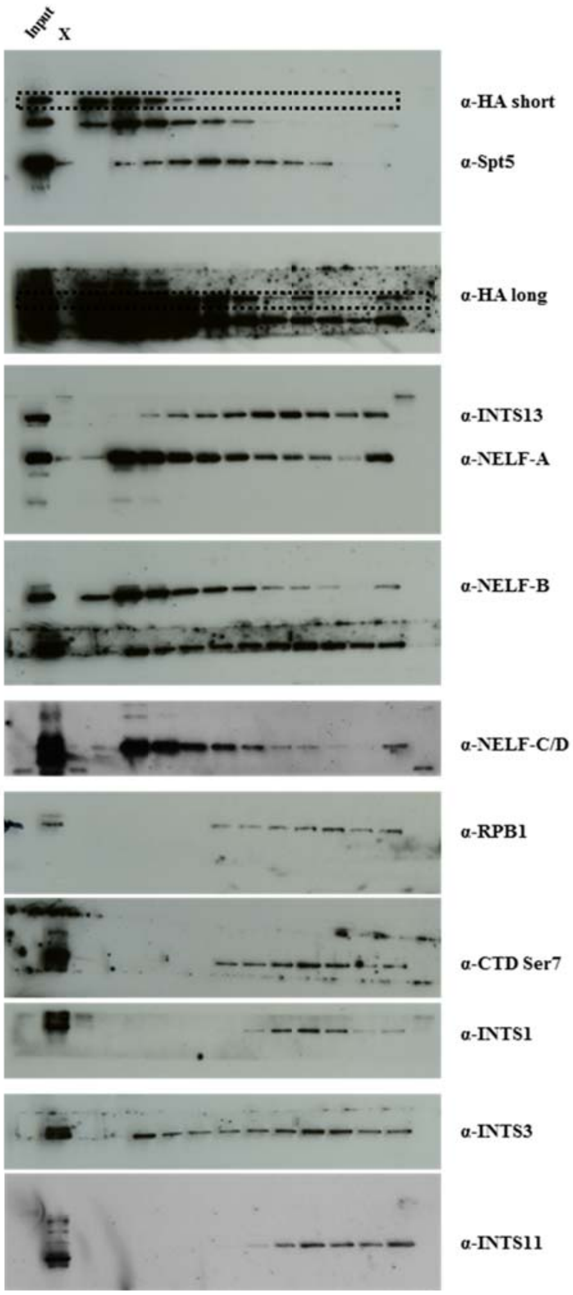

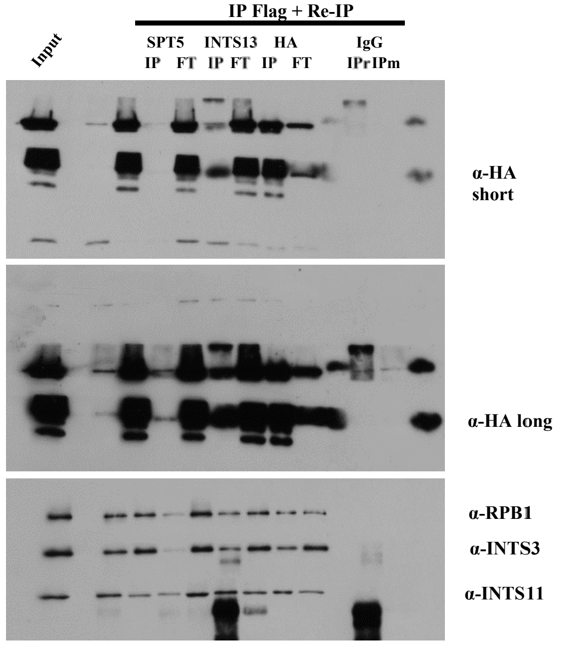

Supplementary Figure 11: Original immunoblot data for Figure 1 and supplementary figure 1A

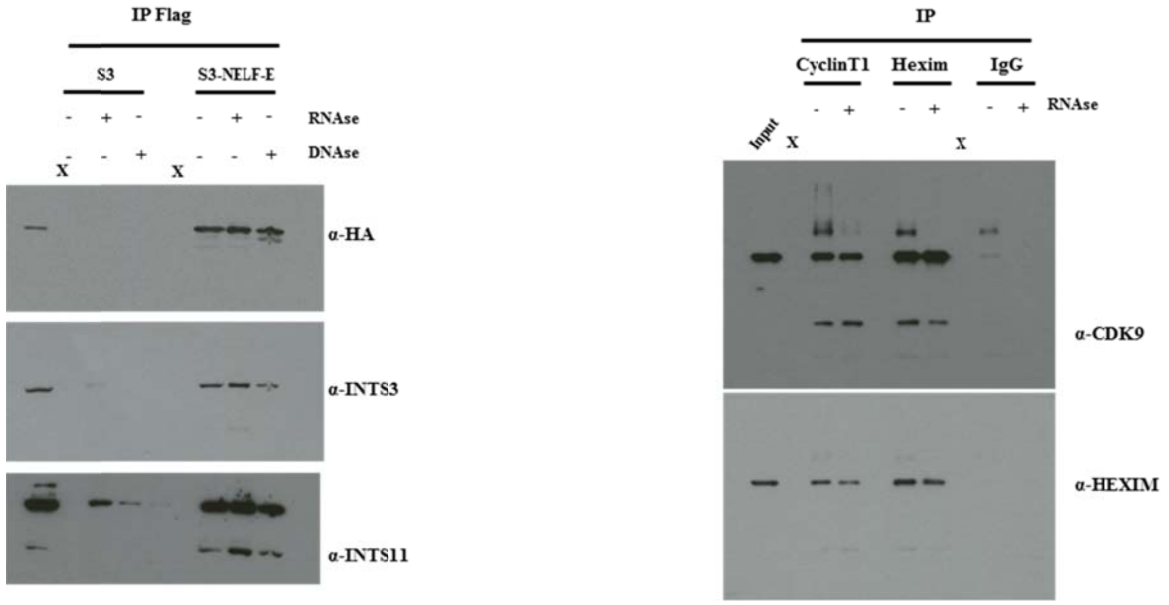

Supplementary Figure 12
Original immunoblot data for supplementary Figure 1B and 1C

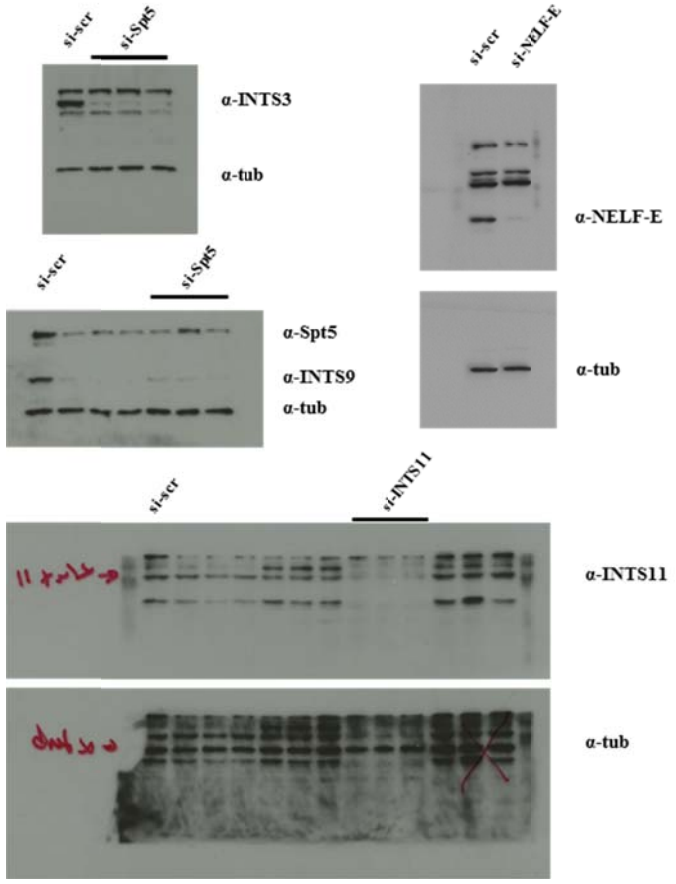

Supplementary Figure 13
Original immunoblot data for supplementary figure 3

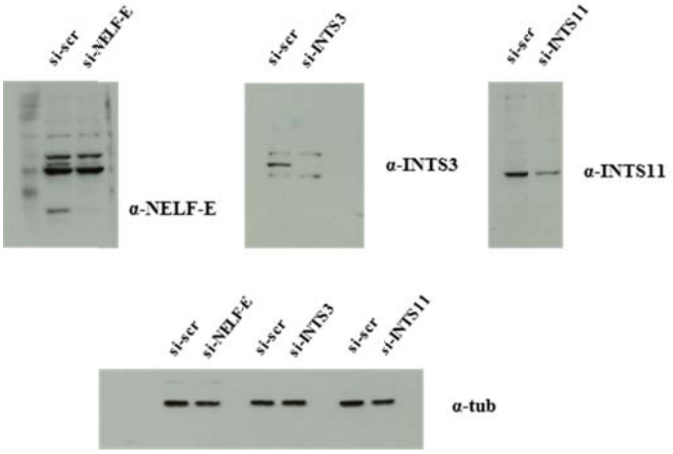

Supplementary Figure 14
Original immunoblot data for supplementary Figure 4

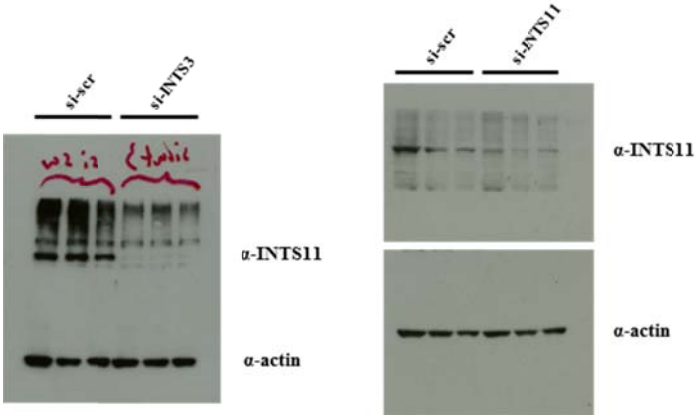

Supplementary Figure 15
Original immunoblot data for supplementary Figure 7

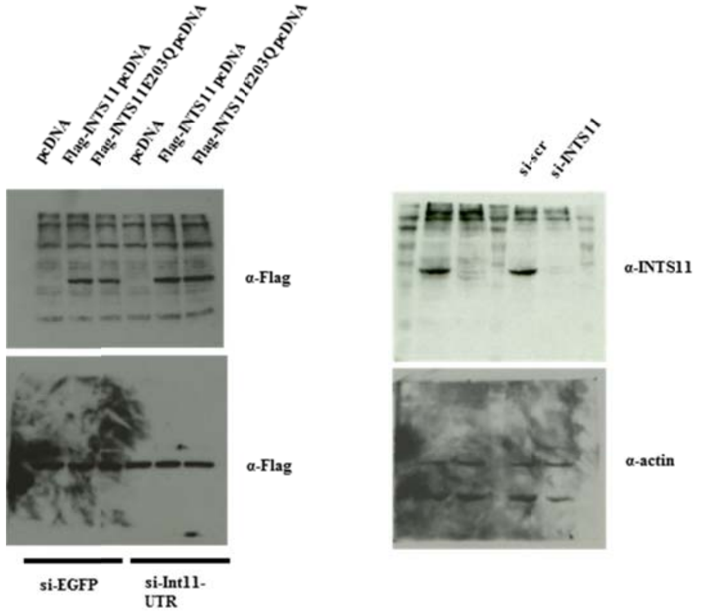

Supplementary Figure 16
Original immunoblot data for supplementary Figure 8

Supplementary Methods

List of primers

HIV1LTR5'-Forward 5'_TCCACTGACCTTTGGATGGT_3'

HIV1LTR5'-Reverse 5'_CTCAGGGTCATCCATTCCAT_3'

HIV1TAR-Forward 5'_GGGTCTCTCTGGTTAGA_3'

HIV1TAR-Reverse 5'_GGGTTCCCTAGTTAGCC_3'

lucMid-Forward 5'_TTCCATTCCATCACGGTTTT_3'

lucMid-Reverse 5'_AGTGCTTTTGGCGAAGAATG_3'

luc3'-Forward 5'_TGGCAGGTCTTCCCGACGAT_3'

luc3'-Reverse 5'_GGCGACGTAATCCACGATCTCT_3'

U2 region 1-Forward 5'_GAGCGGAGCGTTCTCTGTC_3'

U2 region 1-Reverse 5'_CTCCTTGGCCTAGCGGTAAT_3'

U2 region 2-Forward 5'_GATGAGAGTGGGACGGTGAC_3'

U2 region 2-Reverse 5'_CACTTGATCTTAGCCAAAAGG_3'

U2 region 3-Forward 5'_TTCCCTGAAGTACCGTGAGG_3'

U2 region 3-Reverse 5'_CTAAGGACCTCCCCAAAGGA_3'

U2 region 5-Forward 5'_CTCCCTCGCTCTCTCTTTTG_3'

U2 region 5-Reverse 5'_CAAACCTAGACGACTGGTGGA_3'

RPS14-Forward 5'_GGCAGACCGAGATGAATCCTCA_3'

RPS14-Reverse 5'_CAGGTCCAGGGGTCTTGGTCC_3'

KDSR-Forward 5'_AGATGAGTTGGACCCATTGC _3'

KDSR-Reverse 5'_ AAGCCATGAGTTTCCACCAG _3'

PIGB-Forward 5'_ CCAAGCACTTCTGTCTGCTG _3'

PIGB-Reverse 5'_ AACACCCATCTTGCCACTTC _3'

7SK-Forward 5'_CCCTGCTAGAACCTCCAAAC _3'

7SK-Reverse 5'_AAGAAAGGCAGACTGCCAC _3'

List of primary antibodies

Anti-NELF-A A301-910A, Bethyl Laboratories

Anti-NELF-B A301-911A, Bethyl Laboratories

Anti-NELF-C/D 11226-1-AP, Proteintech

Anti-NELF-E ABE48, Millipore

Anti-Spt5 611106, BD Transduction Laboratories

Anti- INTS1 A300-361A, Bethyl Laboratories

Anti- INTS3 16620-1-AP, Proteintech

Anti- INTS3 A302-051A, Bethyl Laboratories

Anti- INTS3 ab70451, Abcam

Anti- INTS9 11657-1-AP, Proteintech

Anti- INTS11 A301-274A, Bethyl Laboratories

Anti- INTS13 19892-1-AP, Proteintech

Anti- INTS13 A303-575A, Bethyl Laboratories

Anti-RNAPII total sc-899, Santa Cruz

Anti-RNAPII Ser2 04-1571, Millipore

Anti-RNAPII Ser5 04-1572, Millipore

Anti-RNAPII Ser7 04-1570, Millipore

Anti-Hexim1 ab25388, Abcam

Anti-Cdk9 sc-13130, Santa Cruz

Anti-cyclinT1 sc-10750, Santa Cruz

Anti-FLAG A2220, Sigma

HA sc-805, Santa Cruz

BrdU sc-32323 AC, Santa Cruz

Supplementary references

1 Egloff, S., Zaborowska, J., Laitem, C., Kiss, T. & Murphy, S. Ser7 phosphorylation of the CTD recruits the RPAP2 Ser5 phosphatase to snRNA genes. *Mol Cell* **45**, 111-122, doi:10.1016/j.molcel.2011.11.006 (2012).

2 Josse, J., Pages, J. & Husson, F. Multiple imputation in principal component analysis. *Advances in Data Analysis and Classification* **5**, 231-246, doi:10.1007/S11634-011-0086-7 (2011).

3 Gilchrist, D. A. *et al.* Pausing of RNA polymerase II disrupts DNA-specified nucleosome organization to enable precise gene regulation. *Cell* **143**, 540-551, doi:10.1016/j.cell.2010.10.004 (2010).
