# Supplementary material for: Integrator complex regulates NELF-mediated RNA polymerase II pause/release and processivity at coding genes
Source: Nat Commun. 2014 Nov 20;5:5531. doi: 10.1038/ncomms6531 (PMC4263189; doi:10.1038/ncomms6531)
Supplement: Supplementary Dataset 1 — Number of unique peptides recovered for eNELF-E associated factors identified by tandem mass spectrometry. [file ncomms6531-s2.ppt]

## Slide 1
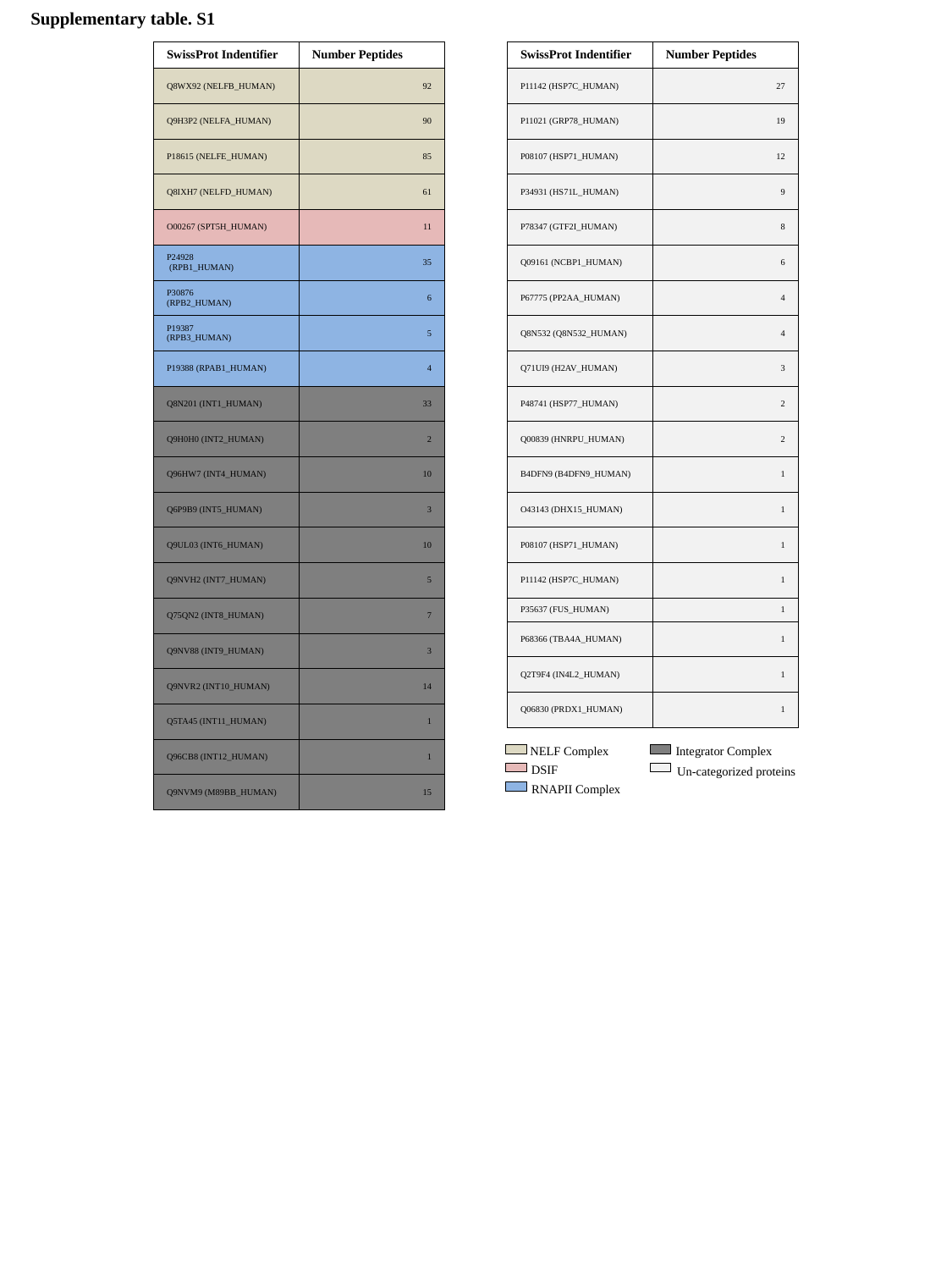

Supplementary table. S1
| SwissProt Indentifier | Number Peptides |
| --- | --- |
| Q8WX92 (NELFB\_HUMAN) | 92 |
| Q9H3P2 (NELFA\_HUMAN) | 90 |
| P18615 (NELFE\_HUMAN) | 85 |
| Q8IXH7 (NELFD\_HUMAN) | 61 |
| O00267 (SPT5H\_HUMAN) | 11 |
| P24928 (RPB1\_HUMAN) | 35 |
| P30876 (RPB2\_HUMAN) | 6 |
| P19387 (RPB3\_HUMAN) | 5 |
| P19388 (RPAB1\_HUMAN) | 4 |
| Q8N201 (INT1\_HUMAN) | 33 |
| Q9H0H0 (INT2\_HUMAN) | 2 |
| Q96HW7 (INT4\_HUMAN) | 10 |
| Q6P9B9 (INT5\_HUMAN) | 3 |
| Q9UL03 (INT6\_HUMAN) | 10 |
| Q9NVH2 (INT7\_HUMAN) | 5 |
| Q75QN2 (INT8\_HUMAN) | 7 |
| Q9NV88 (INT9\_HUMAN) | 3 |
| Q9NVR2 (INT10\_HUMAN) | 14 |
| Q5TA45 (INT11\_HUMAN) | 1 |
| Q96CB8 (INT12\_HUMAN) | 1 |
| Q9NVM9 (M89BB\_HUMAN) | 15 |
| SwissProt Indentifier | Number Peptides |
| --- | --- |
| P11142 (HSP7C\_HUMAN) | 27 |
| P11021 (GRP78\_HUMAN) | 19 |
| P08107 (HSP71\_HUMAN) | 12 |
| P34931 (HS71L\_HUMAN) | 9 |
| P78347 (GTF2I\_HUMAN) | 8 |
| Q09161 (NCBP1\_HUMAN) | 6 |
| P67775 (PP2AA\_HUMAN) | 4 |
| Q8N532 (Q8N532\_HUMAN) | 4 |
| Q71UI9 (H2AV\_HUMAN) | 3 |
| P48741 (HSP77\_HUMAN) | 2 |
| Q00839 (HNRPU\_HUMAN) | 2 |
| B4DFN9 (B4DFN9\_HUMAN) | 1 |
| O43143 (DHX15\_HUMAN) | 1 |
| P08107 (HSP71\_HUMAN) | 1 |
| P11142 (HSP7C\_HUMAN) | 1 |
| P35637 (FUS\_HUMAN) | 1 |
| P68366 (TBA4A\_HUMAN) | 1 |
| Q2T9F4 (IN4L2\_HUMAN) | 1 |
| Q06830 (PRDX1\_HUMAN) | 1 |
NELF Complex
Integrator Complex
DSIF
Un-categorized proteins
RNAPII Complex
